# Brain Ketone Bodies in Health, Evolution and Disease

**DOI:** 10.3390/cells15040382

**Published:** 2026-02-23

**Authors:** Pierre Bougnères

**Affiliations:** 1 Laboratoire des Maladies Neurodégénératives, MIRCe Institute, Commissariat à l’Energie Atomique, Fontenay-aux-Roses, 92260 Paris, France; pierre@bougneres.fr; 2 Groupe d’Etudes Thérapeutiques du Diabète et de l’Obésité, GET-DOC, 92370 Chaville, France

**Keywords:** ketone bodies, brain, evolution, ketogenic diet, Alzheimer

## Abstract

Ketone bodies (KBs) are the only energy substrates oxidized by the brain, whose concentration in the circulation can greatly increase when a physiological situation requires it. For example, when an adult human fasts for two days, circulating KBs rise twenty-fold from ~0.1 to ~2 mM. As a fuel, KBs provide the brain with acetyl-CoA that produces ATP or glutamate, notably in certain brain regions. Remarkably, KBs activate the expression of their own cerebral transporters and KB-utilizing enzymes so that circulating levels determine cerebral utilization of KBs. Throughout evolution, the energetic role of KBs has been crucial for the metabolic homeostasis of humans endowed with a large brain and facing unpredictable periods of food shortage. Paradoxically, the brain of modern, regularly fed humans whose ordinary blood KBs are ~0.1 mM, has access to much fewer circulating sources of energy than that of their distant ancestors. KBs can modify certain proteins post-translationally, for example, histones through lysine-butyrylation. KBs could act as short- or long-term epigenetic messengers. These properties of KBs might allow a fetus to directly sense maternal starvation and adapt their cerebral metabolism to this situation, possibly preparing for nutritional constraints in extra-uterine life. KB transcriptional and epigenetic properties could also enable the postnatal organism to retain a molecular memory of its own starvation episodes. No other energy substrate, such as glucose or lactate, has such capacities. Medicine turned its attention to KBs a century ago. Indeed, KBs are the only energy substrates whose circulating levels can be increased, and nutritional interventions can alter them under free-living conditions. This property opens broad prospects for ketogenic diets (KDs) to prevent or rescue neurodegenerative diseases characterized by glucose hypometabolism, notably Alzheimer’s disease (AD). However, KDs have not yet found real medical applications, for reasons that are discussed.

## 1. Ketone Bodies Biochemistry

The two circulating KBs, β-hydroxybutyrate (BHB, H_3_C–CHOH–CH_2_–COO^−^) and acetoacetate (Acac, H_3_C–CO–CH_2_–COO^−^), are weak acids that are both water-soluble and lipid-soluble.

### 1.1. Hepatic Production of Ketone Bodies

The four carbon atoms of KBs originate from circulating free fatty acids (FFAs) (16 or 18 carbon atoms). FFAs are released in the blood by the hydrolysis of triglycerides (lipolysis) that have been stored in adipose tissue during periods of food abundance. Except during prolonged food deprivation, these reserves are immense in humans from the end of intrauterine life to the whole lifespan. FFAs cannot be directly utilized by the brain [1], unlike the heart, skeletal muscles, and kidneys. To become an energy source for the brain, FFAs are taken up by the liver and beta-oxidized into mitochondria to produce acetyl-CoA. Most of this acetyl-CoA is used for ketogenesis, which requires three enzymatic reactions. Acetoacetyl-CoA thiolase first condenses two molecules of acetyl-CoA to form acetoacetyl-CoA, which is condensed with another molecule of acetyl-CoA by the rate-limiting HMG-CoA synthase 2 to form 3-hydroxy-3-methylglutaryl-CoA (HMG-CoA). HMG-CoA lyase cleaves HMG-CoA into acetyl-CoA and Acac. BHB dehydrogenase (BDH1) reversibly converts Acac into BHB. More stable in the circulation than Acac, BHB is the predominant KB produced by the liver. Lipolysis, hepatic β-oxidation, and ketogenesis are crucially controlled by insulin and are activated when environmental conditions, such as carbohydrate deprivation, lower insulin levels [2].

### 1.2. Energetic Utilization of Ketone Bodies by the Brain

KBs cross the endothelial cells of the blood–brain barrier (BBB) via the high-affinity monocarboxylate transporters MCT1 to reach the extracellular compartment of the central nervous system (CNS) [3]. MCT1 is a proton–monocarboxylate symporter that also transports pyruvate and lactate. By regulating KB entry, MCT1 is a major rate-limiting actor of cerebral KB utilization. Within a self-regulated physiological loop, increased blood BHB upregulates endothelial MCT1 expression, thus increasing intracerebral BHB [3,4,5]. MCT1 expression is also increased by reduced glucose availability [3,4]. ^1^H- Magnetic resonance spectroscopy (MRS) studies in adult humans showed that when circulating BHB concentration increases from 0.05 mM (basal level) to 3.2 mM (3-day-fasting), the corresponding extracellular concentration increases from 0.05 mM to 0.98 mM in the CNS [6,7,8]. For comparison, when blood glucose is ~4.7 mM, its intracerebral concentration is ~1 mM [9]. From the extracellular space, KBs enter neurons and axons via MCT2, another high-affinity transporter [3] whose expression is also stimulated by BHB [3,4]. Within neuronal mitochondria, BHB is converted by BHB dehydrogenase (BDH1) into Acac. Succinyl-CoA:3-oxoacid CoA transferase (SCOT, OXCT1 in humans) converts Acac into acetoacetyl-CoA, which is cleaved into two molecules of acetyl-CoA by Acetoacetyl-CoA thiolase (ACAT1). BDH1, SCOT, and ACAT1 are the KB-utilizing—or “ketolytic”—enzymes of brain mitochondria. Following ketolysis, KB-derived acetyl-CoA enters the tricarboxylic acid (TCA) cycle to generate large amounts of ATP through OXPHOS. This ATP is used by neuronal cell bodies, axons—notably the long myelinated axons—and synapses to support their high energy demand [10,11]. For the TCA cycle to use acetyl-CoA derived from ketolysis, sufficient oxaloacetate should be generated by aerobic glycolysis. Without this glucose-derived supply, KBs cannot be oxidized to support ATP production. In addition to ATP generation, the neuronal TCA cycle produces α-ketoglutarate, the precursor of the neurotransmitter glutamate. While glucose is the dominant precursor for the net synthesis of the α-ketoglutarate–glutamate pool, KBs contribute 10% when circulating BHB concentrations are ~3 mM [7]. Via MCT1, KBs also enter oligodendrocytes [3] to be used as efficient precursors for myelin synthesis and for transfer to axons [12]. KBs are minimally used for energy metabolism in astrocytes and microglia, which rely almost exclusively on glucose and lactate as energy sources [13,14]. Although astrocytes can produce KBs and supply them to neurons in vitro under extreme conditions, (reviewed in [15]) this pathway has not been convincingly demonstrated in vivo and remains a minor, largely speculative component of brain metabolic physiology [15].

### 1.3. Ketone Bodies as Signals

MCT1 and MCT2 are crucial proteins whose expression is modified by BHB [3,4,5]. BHB can also act as a signaling molecule by interacting directly with multiple other proteins (reviewed in [16,17,18,19,20]). BHB can be converted into BHB CoA by short-chain acyl-CoA synthetase, and then a lysine acyltransferase can transfer the β-hydroxybutyryl group from BHB-CoA to lysine. This post-translational modification (PTM)—the covalent attachment of β-hydroxybutyryl residues to the ε-amino group of lysine—was first identified in HeLa cells in 2007 [21], and was called lysine β-hydroxybutyrylation (Kbhb) [21]. The proteins that undergo Kbhb are only beginning to be characterized. Studies have mainly concerned the liver, kidneys, and heart, and far less the brain [22,23]. Functional consequences of Kbhb, both for individual proteins and the global proteome, remain largely unknown [24]. Several targets of Kbhb, however, have been identified. As one of the histone PTMs, Kbhb could regulate chromatin accessibility and gene expression. BHB could also inhibit class I histone deacetylases (HDACs) in vitro and in vivo—though not demonstrated in brain cells—resulting in increased histone acetylation at promoters of genes involved in oxidative stress responses [25]. Through histone acetylation, histone Kbhb, histone methylation, DNA methylation, and microRNA expression [26], BHB can act as an epigenetic modifier in the brain (reviewed in [27]). BHB can also affect protein folding, repair, and degradation, ensuring long-term protein stability—effects that have not yet been fully characterized in brain proteins [28]. Globally, BHB might modulate the proteome at transcriptional and post-transcriptional levels, thus altering protein expression and function [27,29,30,31,32,33]. However, studies of the signaling role of BHB are preliminary and rely on models of questionable relevance, so their findings should be interpreted with caution.

Of particular interest is the ability of BHB to act on epigenetic effectors, as a crucial link between nutritional environment, metabolic status and specific functions of body tissues, notably the brain. Thanks to BHB, the brain might retain epigenetic marks that serve as cerebral memories of periods of food scarcity. This might apply to the fetal brain. Indeed, by crossing the placenta, the KBs of a fasting mother could inform the fetus and prepare him for postnatal life. The duration of such a putative effect is unknown. In the short term, the KB-induced increase in MCT1 and MCT2 expression can help the neonatal brain to make use of KBs. The effects of BHB-induced histone butyrylation could last as long as the histone itself is stable. In addition, BHB-induced changes in DNA methylation, if they occur, could have long-lasting effects. Such epigenetic memory of fast-induced hyperketonemia can also be triggered by food shortage in postnatal life. The epigenetic signaling of BHB appears as a unique mechanism because the other energetic substrates, lactate and glucose, cannot generate epigenetic signals in physiological conditions. Histone PTM (lactylation) and its effect on DNA methylation exist for lactate, but only occur when cells are exposed to supra-physiological lactate concentrations [34].

Another signaling effect of KBs is to modulate glutamate release through allosteric regulation of the vesicular glutamate transporter [35], which reduces glutamate and GABA levels in cortical areas [36,37]. BHB also binds and activates the GPR109A (HCAR2) receptor [38], which inhibits NLRP3 inflammasome activation in microglia, thereby reducing the release of pro-inflammatory cytokines [39]. In addition to these microglial effects, emerging evidence indicates that GPR109A activation can support endothelial barrier integrity and broader neuroprotective functions, although direct evidence for its role, specifically at the BBB, remains limited [40].

## 2. Ketone Bodies Physiology in the Modern World

KB physiology is addressed in this section following the order of life as it passes from birth towards adulthood and old age. Each situation will be characterized by KB circulating concentrations and, whenever possible, dynamic KB fluxes and metabolism.

### 2.1. Birth, Infancy and Childhood

Fetal KB metabolism will be discussed during pregnancy in the next paragraph. In developed societies, newborns are born under comfortable conditions. However, though they are fed with regular milk intakes (100 then 200 mL, i.e., 70–130 kcal daily during the first week), this does not meet their energy demand (~400 kcal/day). This is because the large human neonatal brain (~400 g, i.e., 12.5% of body weight) imposes extremely high energetic costs [41,42] relative to the overall metabolic budget [43]. To supply the brain with enough glucose and KBs during early life, the liver strongly upregulates gluconeogenesis [44] and ketogenesis [45]. KB production is supplied by the large stores of triglycerides (~4000–5000 kcal) [46], generating a massive flux of FFAs, of which approximately 30% are converted into KBs by the liver [47]. Per cell, neonatal hepatocytes produce as many KBs after 4 h of fasting [45] as an adult fasting for 24 h [48,49,50,51,52] [45] (Table 1). A few months later, infants can sustain a 12-h overnight fast thanks to the production of ~15 g KB per 12 h [45] (Table 1). Years later, a 3–5-year-old child who fasts 24 h produces KBs at an average rate of ~50 g/12 h [53], as would do an adult fasting for 3 days [48,49,50,51,52] (Table 1).

Studies of cerebral KB utilization (CMRkb) are scarce in pediatric ages. Arteriovenous measurements showed that at a circulating KB concentration of 1.1 mM, the infant brain extracts ~2 g of KB/12 h, providing ~8% of brain energy, the remainder being supplied by glucose [54]. However, these measurements were performed during surgery under general anesthesia that markedly reduces cerebral activity and fuel utilization. Therefore, while the relative proportions of KBs and glucose utilization are consistent, CMRglc and CMRkb are underestimated by ~50% [54]. Actually, PET imaging in conscious, awake 4-year-old children indicates that CMRglc is ~75 g/12 h [55], much higher than the ~37 g/12 h of the anesthetized brain [56]. PET measurements of CMRkb were not performed in childhood, but extrapolation from glucose suggests that a 4-year-old brain exposed to ~0.5–1 mM circulating KBs would extract ~7.5 g KB/12 h. After 24 h of fasting, with plasma KB at 3–4 mM [57,58], CMRkb could plausibly reach ~23 g/12 h, a rate comparable to that of an adult fasting for 6 weeks [59]. Recall that at 4 years, the brain has almost reached adult volume. During infancy, childhood, and adolescence, the brain accounts for ~44–87% of resting metabolic rate [60], imposing substantial trade-offs with other energy-demanding functions [55,61]. In these ages, the high rate of KB utilization by the brain reflects not only the synaptic activity, but the additional energetic costs associated with the active myelination [62] and the exuberant proliferation of neuronal processes and synapses [63].

The expression of MCT1 and MCT2 transporters increases gradually after 5 months of intrauterine life in humans [64] and after E18 in rat fetuses [65]. No studies of later expression of KB transporters were performed in human infants, while the P14 brain of rat pups showed a massive MCT1 expression in the vessel walls [65]. Ketolytic enzymes are active in the fetal human brain (3–4 months of gestation) [66,67] and remain so in young children [68]. In the brains of rat pups, levels of KB-utilizing enzymes are three-fold higher at weaning than in adult rats [5,69,70]. This pattern of heterochronic expression peaking in the early brain and declining with maturation is shared by rodents and humans. In summary, KBs are as important as glucose for fueling the brain in early life [71] and are also the preferential source of acetyl-CoA for myelin synthesis [72].

Older children retain a high capacity to elevate blood KBs during a ~20-h fast [57,58,73,74,75], compared with adults [48,49,50,51,52] (Table 1). Expectedly, the relative brain mass (~5% at 8 years, ~2.3% at 15 years) gradually reduces the relative cerebral energy demand. KB fluxes, brain MCT transporters, KB-utilizing enzymes, or CMRkb have not been studied in older children or adolescents.

### 2.2. Contemporary Adults

Hepatic KB production in adulthood has been initially estimated by arteriovenous (AV) measurements [49,50,51,52] and by the dilution of ^14^C tracers [48]. These early studies were performed in normal-weight or obese adults in the basal state or during 3–36-day fasting periods, with KB levels varying from ~0.1 mM (basal) to 7.2 mM (prolonged fasting). Basal hepatic KB production was estimated at ~33 g/12 h, increasing up to ~65 g/12 h after 3 days of fasting [49,50,51,52,53].

Using AV differences, Owen and colleagues [59] provided seminal insights into cerebral KB metabolism, showing that in a human fasting for 6 weeks, with an average circulating KB concentration of 7.2 mM, the brain extracts ~18.5 g/12 h of KB and ~21.5 g/12 h of glucose, most of which was oxidized in the TCA cycle, while FFAs were not taken up. About 28% of hepatic KB production was captured by the brain, supplying roughly half of its energy requirements. When asked why a 6-week fasting period was chosen, Owen humorously replied: “Jesus fasted forty days and forty nights; and afterward he hungered” (St. Matthew 4:2) [76]. Cerebral KB uptake was found to be proportional to circulating concentrations [77]. Further studies in overnight-fasted adults (21–65 years) with circulating KB ~0.36 mM demonstrated a cerebral uptake of ~1 g KB and 45 g glucose per 12 h [78]. Such invasive AV experiments have been superseded by non-invasive approaches based on functional imaging of the brain, which have globally replicated the results of AV difference studies. Combined AV difference and FDG-PET studies showed that CMRkb increased from ~2 g/12 h (baseline) to 21 g/12 h after 3 days of fasting, while circulating KBs rose from 0.2 to 2.9 mM [79]. In parallel, CMRglu decreased from 61 to 41 g/12 h. Under these conditions, KBs account for ~25% of cerebral energy requirements [79]. PET using R-β-[1-^11^C]hydroxybutyrate found a low KB uptake under basal conditions (circulating BHB ~0.04 mM), with cortex showing higher utilization than white matter [80]. Acute hyperketonemia increases cerebral KB uptake proportionally to plasma levels, confirming that transport across the BBB is the primary rate-limiting step, and that utilization remains linear at physiological and moderately elevated KB concentrations [80,81]. In ten healthy adults (35 ± 15 y) eating a very-high-fat ketogenic diet (KD) (4.5:1; lipid:protein plus carbohydrates) for 4 days, CMRacac and CMRglc were quantified by PET using ^11^C-Acac and ^18^F-fluorodeoxyglucose (^18^F-FDG) tracers, respectively. Under KD, the circulating KB level increased 8-fold while glucose decreased by only 24%. CMRacac increased 6-fold, whereas CMRglc decreased by 20%. After 4 days on KD, CMRacac represented 17% and total CMRkb ~33% of whole brain energy requirements, with a 2-fold difference across brain regions (12–24%) [82]. In the occipital lobe, ^1^H MRS [83] showed mean brain BHB increasing from 0.05 (non-fasting) to 0.60 mmol/L after 2 days of fasting, and to 0.98 mmol/L after 3 days, correlating well with plasma BHB (0.03, 1.7, and 3.2 mM, respectively) [8]. In response to BHB infusion, circulating BHB rose to 2.1 mM, while brain BHB reached only 0.24 mM [6]. During fasting, the blood-to-brain gradient for BHB was 2.8 (1.7/0.60) while it was 8.8 (2.1/0.24) with acute infusion. The comparison of brain-to-blood ratios shows that brain extracts KBs from blood with a much higher efficacy after a sustained than an acute experimental hyperketonemia [84,85].

Three physiological circumstances—physical activity, pregnancy and lactation—place energy metabolism under high energy demand.

During exercise, the extra KBs formed serve as a fuel for respiration and do not accumulate. After cessation of exercise, the raised concentration of FFAs results in a high rate of KB production (post-exercise ketosis) [86,87]. In overnight-fasted adults, post-exercise KB levels are 0.20 to 2 mM depending on intensity and duration of physical activity [87,88].

To support maternal metabolism, placental function, and fetal growth, pregnancy requires additional energy, estimated at ~250 kcal/day at mid-gestation, and ~450 kcal/day in the third trimester [89]. To meet this demand, maternal lipid mobilization increases, with both FFAs and KBs contributing to energy supply [90]. Early studies in a dozen pregnant women at 4–5 months of gestation showed that after an overnight fast, plasma KBs are ~0.52 mM, three-fold those of non-pregnant women [91]. One of the most detailed studies in normal human pregnancy was performed by Bon and colleagues, who measured energetic substrates in maternal venous blood (MVB) and umbilical venous blood (UVB) in 73 pregnant women [92]. These authors found that maternal glucose is higher than fetal glucose, maternal lactate (1.26 mM) slightly lower than the fetal level (1.48 mM) and maternal FFAs three times higher than in fetal blood. Maternal venous KBs (0.24 mM) are significantly lower than those in UVB (0.44 mM), indicating fetal production of KBs in the basal state. After a 3-day fast at mid-gestation, maternal KBs rose to 5.1 mM, and amniotic fluid KB levels increased 30–40-fold over non-fasted levels, reaching KB concentrations of ~4–5 mM, while amniotic glucose was ~40% lower than in non-fasted women [93]. These results indicate that KBs produced by the mother during caloric deprivation cross into the fetal compartment and accumulate in the amniotic fluid, exposing the fetus to elevated KBs that can contribute to fetal energy metabolism, brain development, and signaling to the fetus during the periods of maternal caloric restriction.

Lactation imposes an additional high energy expenditure of ~500 kcal/day for the mother [89]. In a controlled study of exclusively breastfeeding women undergoing a 42-h fast between 6 and 12 weeks postpartum, plasma BHB rose to 4.3 mM with glucose at 2.6 mM compared with 2 and 3.6 mM, respectively, in non-lactating women [94]. Therefore, KB metabolism becomes particularly active in lactating mothers in response to prolonged fasting or negative energy balance [95].

### 2.3. Brain Ketone Bodies in Elderly Adults

While ketosis induced by prolonged fasting has been extensively characterized in young and middle-aged adults, studies in elderly individuals are scarce. Available data in older adults are limited to short fasting periods, and the effects of multi-day fasting on KB production and utilization in the elderly remain essentially undocumented. After overnight fasting, circulating BHBs are at ~0.12 mM in men or women aged 70–80 years, comparable to younger adults under similar conditions [96]. Elderly humans show a two-fold increase if fasting is prolonged 18 h [97]. When given a ketogenic meal, older adults (~76 years) exhibit BHB increases comparable to young adults (~23 years), and cumulative BHB oxidation did not differ significantly between these age groups [98]. In comparison with younger adults, 74-year-old adults had a mean 8% lower CMRglc in gray matter, specifically in several frontal, temporal, and subcortical regions, and in the cingulate and insula [99]. Age had no effect on CMRacac in gray matter, which did not reach significance. Rate constants of glucose and Acac were significantly lower (−11 and −19%, respectively) in older adults. The Acac index, which expresses the scaled residuals of the voxel-wise linear regression of glucose on KB uptake, identified regions taking up higher (caudate) or lower amounts of Acac relative to glucose [99]. This study provided new information about glucose and KB metabolism and a comparison of the extent to which the regional use of these two substrates changes during normal aging [99].

## 3. Ancient Times and Evolutionary Context

We have no knowledge of the dietary patterns of our distant ancestors. However, we can speculate on how KB metabolism has operated in a prehistoric context, based on the physiological knowledge acquired from studies in contemporary humans. Again, our review will follow a life-course perspective, discussing the potential roles of KBs from fetal life to adulthood. At all stages of life, we will encounter the unexpected paradox that the brain of our ancestors, although exposed to a more erratic feeding, had access to more circulating energy substrates than the brain of regularly fed modern humans.

### 3.1. Fetal Ketone Bodies May Be a Cue for Prediction of Postnatal Metabolic Life

**Figure 1:**
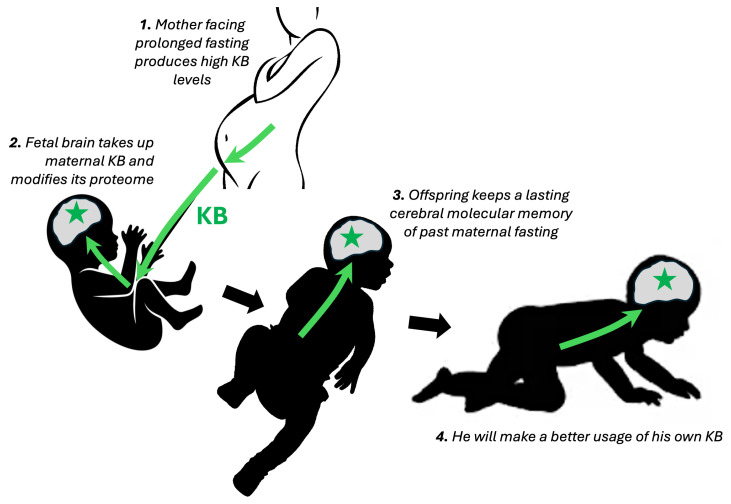
Our hypothesis is that KBs could also act as epigenetic signals to control postnatal expression of specific brain proteins and have a signaling function as a profitable cue for future extrauterine metabolic life. The maternal KB response to hostile nutritional conditions is transmitted to her fetus, whose brain becomes prepared for difficult environmental conditions. Mother’s milk being insufficient for meeting the energy demand, the newly born infant needs to produce their own KBs. If their brain has set an accelerated KB transport and KB-utilizing machinery at the end of gestation, this will help the cerebral energy supply.

In utero, maternal KBs nourish the human fetal brain [66,67]. For our female ancestors living in the wild, meeting the caloric demand of the fetal brain relied on an accelerated KB production and transplacental transfer. The prehistoric fetal brain could therefore draw on a total of ~5–10 mM of circulating maternal energy substrates (sum of KBs and glucose), whereas the brain of a modern fetus has only access to ~5 mM. As in other placental mammals, human females have evolved mechanisms that help buffer the fetus against short-term fluctuations in maternal diet and energy status [100] (see previous paragraph on pregnancy metabolism). Maternal KBs can be utilized for providing energy to the fetal brain during the obligate periods of fasting [66,67], and for activating their own cerebral utilization via MCT transporters and KB-utilizing enzymes. KBs could also act as epigenetic signals to control postnatal expression of specific brain proteins and have a signaling function as a metabolic cue for future extrauterine life (Figure 1). They can contribute to Bateson’s hypothesis that “…human development may involve induction of particular patterns of development by cues that prepare the developing individuals for the type of environment in which they will be likely to live.” [101]. Data from animal models indicate that prenatal and early-postnatal events can initiate long-term changes in the expression of the genetic program that persists much longer in the offspring’s life [102]. The assumption that the fetus has access to a reliable cue of future nutritional conditions is yet untested [103], but is highly plausible. Maternal KBs might provide such a cue. Indeed, KBs could imprint metabolic effectors in the fetal soma, such as brain MCT1, MCT2 or KB-utilizing enzymes. Nongenetic inheritance could also be involved in intergenerational effects through the effect of maternal KBs acting on offspring development in utero [104]. We speculate that KBs might give rise to epigenetic marks that could persist for a variable period to influence postnatal gene expression of certain metabolic genes (Figure 1). According to this hypothesis, a nutritionally restrictive maternal environment might better prepare a fetus to handle future nutrient restriction [105]. The shaping of later life traits by early life environment, known as ‘developmental plasticity’, has been well-documented in humans and animals [106].

### 3.2. Childhood in Ancient Times and Evolutionary Context

Encephalization imposed metabolic constraints on hominid evolution [42,43,107]. Developmental plasticity provides individuals with the flexibility to adjust their trajectory of development to match their environment [103,108]. The brain is a central player in this metabolic plasticity [55]. The metabolic survival of prehistoric infants and children was severely challenged by their hostile environment, because their brain volume and energy demand were roughly the same as today [109]. During the first months and years of life, the caloric intake of prehistoric children depended on breast milk, which had to be produced under harsh environmental conditions. Only in Neolithic times did Homo sapiens children benefit from cow’s milk for those able to digest lactose [110]. Adipose tissue reserves of FFAs allowed young humans to survive postnatal metabolic challenges, supplying their brains with large amounts of KBs [111]. The capacity to activate ketogenesis at birth was already present in Old World primates [112,113], as well as in mice and rats [114]. This was therefore not a recent acquisition of the early humans. Again, in ancient times, the brain of a fasting child could draw on a total of approximately 5–10 mM of circulating energy substrates (KB + glucose), much more than the 5 mM brain of a well-nourished modern child.

### 3.3. Prehistoric Adults

Supposedly, the Paleolithic diet of Homo sapiens relied on animal-based protein-rich foods (19–35% of energy) coupled with the relatively low carbohydrate content of wild plant foods (22–40% of energy) [115], as observed in current hunter–gatherer societies [116,117]. In contrast, the modern Western diet consumed by Homo economicus is composed of an excess of energy-rich refined carbohydrates [115,118,119]. In Paleolithic times up until the agricultural revolution at the onset of the Holocene epoch (~11,000 years ago), environmental fluctuations forced our ancestors to develop a flexible metabolism to adapt their energy needs to various climate, seasonal and vegetation conditions. Metabolic flexibility had to be the rule for safeguarding human survival independent of food availability. Paleolithic humans were repeatedly exposed to unpredictable fasting periods—ranging from a few days or even weeks—between hunting episodes or during seasons of shortage. Based on studies in modern humans, one can imagine the metabolic profiles of our distant ancestors. During fasting, their circulating glucose was likely maintained at ~3.5 mM, to which 4.0–7.0 mM of KB were added, yielding a total circulating fuel concentration of ~8–11 mM. This supply of circulating energy fuels helped the brain preserve alertness to escape predators and cognitive performance despite prolonged starvation. Fasting women develop ketosis more rapidly than men [58], an adaptation essential for meeting the metabolic challenges imposed by the demands of reproduction (pregnancy and lactation) [89,120] in addition to repeated and unpredictable fasting. Modern studies of pregnancy energetics have shown that the total energy cost of supporting pregnancy maintenance, fat deposition, and conceptus demand varies remarkably across modern populations, from a high of 125,000 kcal in well-nourished Swedish women to a low of −7000 kcal in un-supplemented Gambian women [121].

To ensure survival during periods of starvation and easily switch to fat and KB oxidation, certain genes may have evolved to regulate utilization of fatty fuel stores. Such genes were termed “thrifty genes” in 1962 [122]. Furthermore, convincing evidence shows that this ancient genome has remained essentially unchanged over the past 10,000 years and certainly not changed in the past 40–100 years. In modern times, humans switched their primal lifestyle towards a constant availability of energy-dense foods and a lack of exercise [123]. We contend that the combination of continuous food abundance and physical inactivity of Homo Economicus eliminates the evolutionarily programmed homeostatic mechanisms emanating from feast-famine and physical activity-rest cycles. Again, the brain of a fasting and exercising prehistoric adult could draw on a total of approximately 7–10 mM of circulating fuels (sum of KBs and glucose), whereas the brain of a modern human spends most of his life with no more than a total fuel of 5 mM.

## 4. Animal Models of Ketone Bodies Physiology: Rodents and Hibernators

Physiological data for the human species are so extensive that our review gives only limited space to rodent models. Indeed, rodent brain is dominated by olfactory and subcortical structures, while human brains are dominated by an expanded, folded cerebral cortex. Human adults also have a much larger brain-to-body weight ratio (~2%) than rodents (0.5–1%). Laboratory rats and mice are sedentary and regularly fed animals, in contrast to their lives in their natural wildlife environment. Cerebral KB metabolism has been studied in rodents of all ages. We cite herein some of the most informative ones. The effects of fasting or a KD have been studied in rats and mice. Rats offer the advantage of a larger size, which facilitates experimentation, particularly for metabolic brain imaging. However, the translation of these experimental data to human brain KB physiology should be cautious, given the evolutionary distance between the two species.

### 4.1. Rats

In fetal rats [66], as in human fetuses [124], the brain is well equipped with KB-utilizing enzymes. In suckling rat pups, the level of CMRkb is debated: it was found to be higher than in adult rats [71,125]. The expression and activities of the three KB-utilizing enzymes increase between 10 and 20 days postnatally, followed by a steep decline after weaning [5,69,126,127]. This was also observed for MCT1 and MCT 2 [5,65,128,129]. On the basis of blood KBs in suckling rats and the Vmax and Km values for the brain enzymes BDH1 and ACAT1, the capacity of the enzymes was several times greater than the actual rate through these enzymatic steps in vivo [130]. This suggested that entry of KBs into the brain is restricted, in suckling rats, and that metabolism is substrate-limited as in adult rats [131].

Several studies used functional imaging of the adult rat brain to show that fasting for 1.5–2 days induces a large increase. In 36-h-fasted adult rats, spatially localized 1H-13C- MRS found that KB oxidation by the brain cortex was 22 µmol/100 g·min, with estimates up to ~30–43 µmol/100 g·min under conditions of high plasma KB (9.4 mM) [132]. In other adult rats, PET imaging with ^11^C-Acac showed that CMRkb exhibited more than a two-fold increase with 48-h-fasting or experimental ketonemia [84]. This effect was mirrored in another PET study, where ^11^C-Acac uptake in the intact rat brain increased by seven- to eight-fold on either a ketogenic diet or following 48 h of fasting relative to a high-carbohydrate control diet, providing in vivo evidence that CMRkb in the adult rat brain is sensitively upregulated in parallel with circulating KBs and fasting conditions [133]. However, comparison with human data is complicated by species differences in methodology, regional measurements, and physiological context [84,134]. To investigate if CMRglc decreases with chronic ketosis, 2-^18^F-FDG in combination with ^11^C-acetoacetate PET was applied in anesthetized young adult rats fed 3 weeks of either standard or ketogenic diets. CMRacac increased approximately 7- to 8-fold under fasting or ketogenic diet, raising plasma KB (~1.5 mM), while CMRglc significantly decreased by ~30% in the cerebral cortex and cerebellum [133].

Studies in rats indicate that different brain regions oxidize KBs with differing efficiencies. An autoradiographic study of the adult rat brain using 3-^14^C-BHB showed that some regions had very limited labeling, notably the basal ganglia [71]. The authors hypothesized that BHB metabolism in brain regions is limited by permeability; areas known to have no BBB show the highest rate of utilization. The results imply that rather than substitute fuels, KBs should be considered supplements that partially supply specific areas but do not support the entire energy requirement of all brain regions. Such regional differences were not found by Cremer [130,135] in the 21-day-old rat brain. This probably emphasizes a variation in the BBB transport process across different ages. Under conditions of ketosis, glucose consumption is decreased in the cortex and cerebellum by about 10% per each mM of plasma KB [136,137]. Rat gestation is associated with hyperketonemia [138,139,140,141].

### 4.2. Mice

The term “ketogenic diet” (KD) broadly refers to a range of dietary regimens that vary across studies, leading to highly variable elevations of circulating KBs, from 0.8 mM [142,143] to a maximal 5.7 mM [5]. Analysis of several representative studies employing a KD in mice has identified multiple factors that influence KB responses. Mouse strain significantly affects KB production, which is why the C57BL/6 strain was selected in all cited studies due to its robust ketogenic response. Age also plays a role: studied mice ranged from 7 weeks to 22 months, with young mice showing greater KB elevation [5,144,145,146], whereas old mice exhibit only minimal increases [143]. The degree of circulating glucose reduction, which varied from none [142] to 50% [5,144], further modulates the brain’s response to KD-induced hyperketonemia. Other important variables include the duration of KD, which ranged from 10 days to 12 months depending on the protocol, and fasting duration, both during [144,147] and outside [148] of the KD. Time of sample collection (day versus night) affects circulating KB levels, which are 1.3- to 3-fold higher at night [149]. The mode of KD administration—continuous versus cyclical—also influenced outcomes, with KB levels notably higher when the KD alternated with a standard diet [149]. Supplementation of KD with MCT or ketone salt can increase KB levels [144,146]. It would be erroneous to think of the brain as using energetic substrates homogeneously across its anatomical and functional structures. Indeed, the coupling of stable-isotope infusions with MS imaging revealed that metabolism is region-specific [150]. In response to a carbohydrate-rich diet, there is nearly uniform use of glucose to fuel the TCA cycle, while in a KD, BHB contributes most strongly in the hippocampus and least in the midbrain [150]. Consistent with brain areas’ preference for glucose or BHB, the regional use of the two classes of substrates as a glutamate source is inversely correlated on KD [150]. In conclusion, predicting the exact KB and glucose regional response to a given KD is challenging. Therefore, assessment of substrate responses is recommended when studying cerebral energy metabolism and brain functions such as memory and cognition. Heterogeneity in mouse models—including differences in strain, age, sex, genetic background, KD protocols, and blood KB levels—can lead to highly variable metabolic outcomes. This variability should be carefully considered in the design of future experiments, including brain imaging studies. Standardizing metabolic conditions and readouts will improve both reproducibility and translational relevance.

### 4.3. Hibernating Rodents

Because these animals endure several months without food intake, hibernating rodents—ground squirrels [151,152,153,154], hamsters [155] and liver jerboa [156]—have a place in this review as examples of extreme physiological adaptation to prolonged fasting. Indeed, hibernation is a fascinating physiological condition that significantly elevates KB levels. Survival relies entirely on the mobilization of fatty acid stores. Crucially, while most organs can use fat, the brain cannot use fat as its sole source of fuel. Circulating KBs are highest during deep torpor (1–4 mM) in a reciprocal relationship with glucose. The brain facilitates this switch by upregulating MCT1 at the BBB [151,152], which increases the passage of KBs into CNS cells. There is a distinct preference for KB utilization in the brain and heart during torpor. BHB is metabolized via the TCA cycle, while glucose metabolism is minimal. Low glutamate helps keep neuronal activity suppressed [157], creating a highly conserved, low-energy state that protects the brain [158].

## 5. Ketone Bodies as Therapeutic Agents

Remarkably, KBs are the only natural substrates oxidizable by the brain that can be increased in circulation through dietary intervention. Indeed, blood glucose or lactate cannot be durably increased in free-living conditions. We have previously seen that in animal models, KDs encompass a variety of nutritional protocols. Since not all KDs are created equal, we review herein the various strategies used for increasing KB levels in humans.

### 5.1. Prolonged or Intermittent Fasting

Fasting is distinct from caloric restriction, in which the daily caloric intake is reduced chronically by 20–40%, but meal frequency is maintained [159] and from intermittent fasting, an eating pattern that alternates periods of food intake with complete fasting [160]. It somewhat mimics the dietary life of our distant ancestors, who spent their lives under a regimen of repeated, prolonged fasting episodes. Repeated cycles of 1–2 days of fasting could be used to induce hyperketonemia as a preventive strategy for neurological diseases in aging individuals. However, this regimen cannot be implemented under contemporary living conditions constrained as is Homo economicus by their meal-eating habits. Incorporating KDs into clinical trials for patients with established neurological conditions would also be extremely difficult. In addition, financial support for low-cost, non-pharmacological trials would have to rely almost exclusively on public research funds.

### 5.2. Classical Ketogenic Diet

The most frequently studied KD in the medical and scientific literature is based on a simple principle—a severe restriction of dietary carbohydrate. Herein, we call it “classical KD” for clarity. In classical KD, carbohydrates contribute only to ≤1% of calories and are replaced by 80–90% lipids for maintaining an isocaloric intake, and relatively low protein content. The deprivation of carbohydrate feeding has multiple hormonal and metabolic consequences. Prominently, it abolishes insulin secretion, which has two consequences. First, it triggers the chronic mobilization of triglycerides from adipose tissue, which is accelerated, releasing a large flux of FFAs into circulation. Second, the very low insulin level stimulates the intrahepatic conversion of a large proportion of these FFAs into KBs. Circulating KB levels reach high—though variable—concentrations depending on the protocol and species, while blood glucose level decreases.

Since KD was conceived more than a century ago, at a time when the metabolism of KB was only beginning to be understood, it is interesting to briefly recall the historical context of this discovery—an achievement initiated outside the academic world. In the 1910s–1920s, B. Macfadden popularized fasting in his magazine—*Medical Culture*—read by hundreds of thousands of Americans [161]. Following his ideas, H.W. Conklin, an obscure osteopath from a small Michigan town, began treating epileptic patients by having them fast for several days [162]. The news of his ~50% successes spread beyond Michigan [163,164] and prompted H.R. Geyelin at the Presbyterian Hospital in New York to deprive epileptic patients of food for 20 days. He reported his results at the American Medical Association convention in 1921 [165], but his extensive clinical data were never published. He later told that long-term freedom from seizures occurred in 15 of 79 children treated (19%), but in only 1 of 200 adults (0.5%) [163,166]. As early as 1914, it had been reported that 3-day starvation and a fat diet were associated with high levels of urinary KBs [167], as later confirmed in a 1921 review article [168]. This observation prompted R.M. Wilder at the Mayo Clinic to propose “that the benefits of fasting could be obtained by provoking ketogenesis with diets which are very rich in fat and low in carbohydrate [169]. Wilder coined the term “ketogenic diet” [170] and described the dramatic improvement in seizure control of three patients with epilepsy [170]. Between 1924 and 1930, a handful of American pediatricians acted on Wilder’s suggestion with a diet composed of one gram of protein per kg of body weight, 10–15 g of carbohydrate per day, and the remainder of the calories in fat, identical to the KD that is used today [171]. These initial reports were rapidly followed by reports at the Massachusetts General Hospital [172,173,174,175,176,177,178]. In 1927, Talbot and Helmholz separately reported that the KD freed 46 out of 144 (31%) epileptic children from attacks and improved an additional 23% (34/144) [172,175,177]. However, by 1937, the KD was found difficult, rigid, and expensive [179], and progressively abandoned.

### 5.3. Medium-Chain Triglycerides (MCT) Supplementation

MCTs are made of medium-chain fatty acids (MCFAs)—octanoate (C8), decanoate(C10), and dodecanoate (C12)—esterified by glycerol. MCFA are present in the milk of mammalians including humans (~15% of total fatty acids) [180]. Ingested MCTs are hydrolyzed in the intestinal lumen into glycerol and medium-chain fatty acids (MCFAs). Glycerol is a gluconeogenic substrate that contributes actively to hepatic glucose production [47,181]. MCFAs are absorbed by enterocytes and transported via the portal vein to the liver for KB production or release in the general circulation. Unlike LCFA, MCFA are not a significant component of adipose reserves. They have two effects on energy metabolism. First, they fuel hepatic ketogenesis without the need for a carnitine-dependent mitochondrial transport [182]. MCFA can also be directly utilized by the brain, because they cross the BBB freely and are taken up by astrocytes and converted locally into small amounts of KBs to supply neurons (brain ketogenesis) [144,183]. Two different dietary strategies make use of MCT. They can be incorporated into the classical KD described above. They can also be given alone without an associated severe carbohydrate restriction. However, this option increases circulating KBs to only a 0.5 mM level [184]. MCFAs also have the property of stimulating the secretion of glucagon-like peptide-1 (GLP-1) [185,186], which has receptors in the brain [187].

### 5.4. Ketogenic Chemicals

A few exogenous chemicals, including ketone esters [188,189,190,191] and butanediol [192,193], initially developed to feed astronauts [194] or glycerol tri-acetate [195], induce various degrees of hyperketonemia without carbohydrate restriction. An ongoing trial is currently testing a ketone monoester in AD patients [196].

## 6. Brain Ketone Bodies in Neurological Diseases

KDs can be applied at different stages of life for the prevention or rescue of an already advanced disease. Listing all the diseases for which KDs have been tested and commenting on the results of these attempts is beyond the scope of this review, because it would require reviewing a total of 2808 publications with 88,119 citations between 2000 and 2021 [197]. KDs have been most widely studied in neurological disorders such as epilepsy [198,199], Parkinson’s disease [189,200,201], multiple sclerosis [202], migraine [203], traumatic brain injury [204,205], stroke [206], and psychiatric disorders [207,208,209]. Globally, existing studies are generally limited by small sample sizes, short intervention durations, heterogeneous patient populations, and variability in dietary protocols. Most trials primarily assess cognitive outcomes or surrogate biomarkers rather than direct measures of disease progression or neuropathology. Therefore, the impact of ketogenic interventions on neurodegenerative processes remains largely uncertain. Accordingly, the overabundant clinical literature has so far not led to a recognized, validated treatment prescribed by clinicians. Because the most promising effects are in the domains of cognition and Alzheimer’s disease, the last chapter of this review will focus on cognition in aging people and AD.

### 6.1. KB Metabolism, Cognition, and Normal Aging

Population aging is the most important social and medical demographic issue worldwide; therefore, healthy aging is important. PET and functional magnetic resonance imaging (fMRI) have been used to explore the functional neuroanatomy of brain energy metabolism and cognitive functions of cognitively healthy aged individuals [210], showing that reduced GLUT1 expression at the BBB and increased brain insulin resistance synergize to limit neuronal glucose availability and metabolism [211]. Early PET work showed that CMRglc decreases with age across frontal, temporal, and cingulate regions [99,212]. In contrast, CMRacac is relatively preserved [99,212]. Converging evidence from fMRI supports that alterations in brain energy supply are linked to changes in cognitive acuity. Using large fMRI datasets, “network stability” was established as a measure of the robustness of functional communication between brain regions. [213]. This biomarker declines with age and correlates with reduced cognitive performance across the entire adulthood [213]. In adults aged < 50 y, KDs stabilized network communication compared with a glucose-dominant state, suggesting that KB utilization enhances functional brain dynamics linked to cognition [213]. A PET study using ^11^C- Acac and ^18^F-FDG comparing 4- and 21-month rats found higher FDG uptake in the hippocampus of aged rats, and a lower percentage contribution of Acac to total brain energy substrate uptake, but cognitive performance was not evaluated [214]. Vice versa, a study found that 22-month-old rats had improved cognitive performance after a 3-week KD, but brain KB or glucose metabolism was not quantified [215]. Although extending rodent cognitive findings to human neurodegeneration is risky [216], these observations point to a coherent model: increasing cerebral KB availability may buttress neural network function, thus supporting cognitive aging. However, direct PET studies in cognitively normal old adults that combine metabolic imaging with neuropsychological evaluation under ketogenic conditions are lacking. Bridging this gap would substantially strengthen causal inferences about the role of KBs in normal cognitive aging.

### 6.2. Alzheimer’s Disease (AD)

The increasing prevalence of dementia and cognitive decline is a major health concern. In 2019, AD affected 5.8 million Americans at a cost of $209 billion USD, and will reach an estimated 14 million Americans and $1.2 trillion USD in 2050 [217]. Since the early 1980s, impaired glucose supply to or metabolism by the brain has been associated with AD risk or progression [212,218,219,220]. Glucose hypometabolism—most notably in the parietal cortex, posterior cingulate, precuneus, thalamus and hippocampus—is a well-established feature of AD, as evidenced by PET/MRI studies reporting a 13–21% reduction in the CMRglc of brain gray matter [212,221,222,223]. Whether brain glucose hypometabolism is a primary driver or a consequence of AD remains unresolved as a typical “chicken-or-egg” conundrum. Indeed, causality cannot be inferred from correlation alone, but requires that causes precede effects [224]. Some studies and opinion reviews suggest that glucose hypometabolism may precede the typical neuropathology of AD [212] while CMRkb appears largely preserved, both globally and regionally [221,225,226]. In this respect, it is interesting that the hippocampus of KD-treated mice uses KBs avidly [150]. Epigenetic processes, possibly triggered by KBs, may link ketogenic interventions to the protection of the brain against age-related pathologies [227]. Dozens of publications, including systematic reviews, meta-analyses, clinical studies, randomized controlled trials and narrative reviews, have examined the effect of KDs in AD [228,229,230,231]. Although study designs and outcomes were heterogeneous, reviews contend that these nutritional interventions may improve cognition in AD [228,229,230,231]. Notably, Cunnane et al. have repeatedly reinforced the concept of KBs as not merely a backup fuel [77,78,79,80], but a preferred substrate for the brain [212]. However, the interpretation of existing trials is complicated by limited sample size, lack of randomization, short duration, questionable dietary adherence, and disease stage. Importantly, a symptomatic relief in cognitive impairment does not mean that AD progression is being altered by KDs. KD-induced changes in regional brain glucose and KB uptake are poorly understood. Notably, PET studies of KB utilization by the brain have not been associated with a clinical benefit. Clinical recommendations will thus await the results of larger and long-term trials. Patient subgroups most likely to benefit will also have to be identified. This will require therapeutic trials integrating clinical assessment, neuroimaging [61,232], biomarkers [233,234], alongside machine learning techniques, to target high-risk patients or patients with early prodromal AD [231]. It should also be considered that if KD was to be given for AD prevention in the general aging population, there would be some concern regarding potential long-term unwanted effects, particularly with respect to cardiovascular health [235].

## 7. Conclusions

KBs are powerful energy substrates for the brain, particularly during early life. Remarkably, they are the only natural substrates oxidizable by the brain that can be increased in circulation through dietary interventions, unlike glucose or lactate. The direct and indirect effects of BHB on the cerebral proteome, including epigenetic modulation, are actively being explored. As early as the intrauterine period, we speculated that BHB may contribute to the molecular “memory” of maternal fasting episodes by priming the fetal brain (Figure 1). Indeed, based on KB’s role as the major fuel of the brain, KDs have been shown to modulate cerebral metabolism. Various KDs have been tested in neurological disorders. To date, evidence largely points to symptomatic cognitive benefits rather than clear effects on neuropathological mechanisms or disease progression. While these findings are promising, future trials will need a stronger pathophysiological foundation to establish genuine therapeutic relevance.

## Figures and Tables

**Table 1:** KB concentrations, hepatic production rate and cerebral utilization (CMRkb) in physiological situations in humans. Rates are expressed in g per 12 h to allow comparison across ages. Values are approximated for a typical individual based on the multiple references cited in the text.

	Fast	Blood KB (mM)	KB Production (g/12 h)	CMRkb (g/12 h)
6-mo-old Infant	12 h	1.2	15	
4-yr-old Child	24 h	2–4	50	23
Adult	12 h	0.05–0.1	33	1–2
	3 d	3–4	65	20
	6 wk	7		19
Pregnant Woman	12 h	0.4		
	3 d	5		
Lactating Woman	2 d	4		
Elderly Adult	12 h	0.1		
	18 h	0.2		

## Data Availability

No new data were created or analysed in this study.

## Abbreviations

The following abbreviations are used in this manuscript:

Acac	Acetoacetate
ACAT	Acetoacetyl-CoA Thiolase
AD	Alzheimer’s Disease
BBB	Blood–Brain Barrier
BHB	Beta-Hydroxybutyrate
BDH1	3-Hydroxybutyrate Dehydrogenase
CMRacac	Cerebral Utilization of Acetoacetate
CMRglc	Cerebral Utilization of Glucose
CMRkbs	Cerebral utilization of Ketone bodies
CNS	Central nervous system
FDG	Fluorodeoxyglucose
FFAs	Free Fatty Acids
fMRI	Functional magnetic resonance imaging
GABA	Gamma-Aminobutyric Acid
HDACs	Histone Deacetylases
KBs	Ketone Bodies
Kbhb	Lysine-Butyrylation of Proteins
KD	Ketogenic Diet
LCFAs	Long Chain Fatty Acids
MCFAs	Medium-Chain Fatty Acids
MCTs	Medium-Chain Triglycerides
MCTs	Monocarboxylate Transporters
MRS	Magnetic Resonance Spectroscopy
OXPHOS	Oxidative Phosphorylation
PET	Positron Emission Tomography
PTM	Post-Translational Modification
SCOT	Succinyl-CoA:3-oxoacid CoA transferase
TCA	Tricarboxylic Acid Cycle

## References

[1] Schönfeld P., Reiser G. (2013). Why Does Brain Metabolism Not Favor Burning of Fatty Acids to Provide Energy? Reflections on Disadvantages of the Use of Free Fatty Acids as Fuel for Brain. J. Cereb. Blood Flow. Metab..

[2] McGarry J.D., Foster D.W. (1980). Regulation of Hepatic Fatty Acid Oxidation and Ketone Body Production. Annu. Rev. Biochem..

[3] Felmlee M.A., Jones R.S., Rodriguez-Cruz V., Follman K.E., Morris M.E. (2020). Monocarboxylate Transporters (SLC16): Function, Regulation, and Role in Health and Disease. Pharmacol. Rev..

[4] Halestrap A.P., Wilson M.C. (2012). The Monocarboxylate Transporter Family—Role and Regulation. IUBMB Life.

[5] Düking T., Spieth L., Berghoff S.A., Piepkorn L., Schmidke A.M., Mitkovski M., Kannaiyan N., Hosang L., Scholz P., Shaib A.H. (2022). Ketogenic Diet Uncovers Differential Metabolic Plasticity of Brain Cells. Sci. Adv..

[6] Pan J.W., Telang F.W., Lee J.H., De Graaf R.A., Rothman D.L., Stein D.T., Hetherington H.P. (2001). Measurement of Β-hydroxybutyrate in Acute Hyperketonemia in Human Brain. J. Neurochem..

[7] Pan J.W., De Graaf R.A., Petersen K.F., Shulman G.I., Hetherington H.P., Rothman D.L. (2002). [2,4-^13^ C_2_]-β-Hydroxybutyrate Metabolism in Human Brain. J. Cereb. Blood Flow. Metab..

[8] Pan J.W., Rothman D.L., Behar K.L., Stein D.T., Hetherington H.P. (2000). Human Brain β-Hydroxybutyrate and Lactate Increase in Fasting-Induced Ketosis. J. Cereb. Blood Flow. Metab..

[9] Gruetter R., Novotny E.J., Boulware S.D., Rothman D.L., Mason G.F., Shulman G.I., Shulman R.G., Tamborlane W.V. (1992). Direct Measurement of Brain Glucose Concentrations in Humans by 13C NMR Spectroscopy. Proc. Natl. Acad. Sci. USA.

[10] Attwell D., Laughlin S.B. (2001). An Energy Budget for Signaling in the Grey Matter of the Brain. J. Cereb. Blood Flow. Metab..

[11] Harris J.J., Jolivet R., Attwell D. (2012). Synaptic Energy Use and Supply. Neuron.

[12] Simons M., Nave K.-A. (2016). Oligodendrocytes: Myelination and Axonal Support. Cold Spring Harb. Perspect. Biol..

[13] Beard E., Lengacher S., Dias S., Magistretti P.J., Finsterwald C. (2022). Astrocytes as Key Regulators of Brain Energy Metabolism: New Therapeutic Perspectives. Front. Physiol..

[14] Monsorno K., Buckinx A., Paolicelli R.C. (2022). Microglial Metabolic Flexibility: Emerging Roles for Lactate. Trends Endocrinol. Metab..

[15] Rose J., Brian C., Pappa A., Panayiotidis M.I., Franco R. (2020). Mitochondrial Metabolism in Astrocytes Regulates Brain Bioenergetics, Neurotransmission and Redox Balance. Front. Neurosci..

[16] Puchalska P., Crawford P.A. (2017). Multi-Dimensional Roles of Ketone Bodies in Fuel Metabolism, Signaling, and Therapeutics. Cell Metab..

[17] Nelson A.B., Queathem E.D., Puchalska P., Crawford P.A. (2023). Metabolic Messengers: Ketone Bodies. Nat. Metab..

[18] Puchalska P., Crawford P.A. (2021). Metabolic and Signaling Roles of Ketone Bodies in Health and Disease. Annu. Rev. Nutr..

[19] Newman J.C., Verdin E. (2017). β-Hydroxybutyrate: A Signaling Metabolite. Annu. Rev. Nutr..

[20] Mihaylova M.M., Chaix A., Delibegovic M., Ramsey J.J., Bass J., Melkani G., Singh R., Chen Z., Ja W.W., Shirasu-Hiza M. (2023). When a Calorie Is Not Just a Calorie: Diet Quality and Timing as Mediators of Metabolism and Healthy Aging. Cell Metab..

[21] Chen Y., Sprung R., Tang Y., Ball H., Sangras B., Kim S.C., Falck J.R., Peng J., Gu W., Zhao Y. (2007). Lysine Propionylation and Butyrylation Are Novel Post-Translational Modifications in Histones. Mol. Cell Proteom..

[22] Cornuti S., Chen S., Lupori L., Finamore F., Carli F., Samad M., Fenizia S., Caldarelli M., Damiani F., Raimondi F. (2023). Brain Histone Beta-Hydroxybutyrylation Couples Metabolism with Gene Expression. Cell Mol. Life Sci..

[23] Hu E., Du H., Shang S., Zhang Y., Lu X. (2020). Beta-Hydroxybutyrate Enhances BDNF Expression by Increasing H3K4me3 and Decreasing H2AK119ub in Hippocampal Neurons. Front. Neurosci..

[24] Han W., Zhang B., Zhao W., Zhao W., He J., Qiu X., Zhang L., Wang X., Wang Y., Lu H. (2025). Ketogenic β-Hydroxybutyrate Regulates β-Hydroxybutyrylation of TCA Cycle-Associated Enzymes and Attenuates Disease-Associated Pathologies in Alzheimer’s Mice. Aging Cell.

[25] Shimazu T., Hirschey M.D., Newman J., He W., Shirakawa K., Le Moan N., Grueter C.A., Lim H., Saunders L.R., Stevens R.D. (2013). Suppression of Oxidative Stress by β-Hydroxybutyrate, an Endogenous Histone Deacetylase Inhibitor. Science.

[26] Abrego-Guandique D.M., Cione E., Caroleo M.C., Bonilla D.A., Cannataro R. (2025). Ketogenic Diet and microRNAs: Focus on Cognitive Function. Front. Nutr..

[27] He Y., Cheng X., Zhou T., Li D., Peng J., Xu Y., Huang W. (2023). β-Hydroxybutyrate as an Epigenetic Modifier: Underlying Mechanisms and Implications. Heliyon.

[28] Madhavan S., Roa Diaz S., Peralta S., Nomura M., King C., Lin A., Bhaumik D., Shah S., Blade T., Gray W. (2025). β-Hydroxybutyrate Is a Metabolic Regulator of Proteostasis in the Aged and Alzheimer Disease Brain. Cell Chem. Biol..

[29] Brickner J.H. (2023). Inheritance of Epigenetic Transcriptional Memory through Read–Write Replication of a Histone Modification. Ann. N. Y. Acad. Sci..

[30] Cheng S., Cheng C., Zhou J., Ji Y., Liu Y., Jiang W., Liu C., Wang H. (2025). Metabolism Meets Epigenetics: β-Hydroxybutyrate–Driven Lysine β-Hydroxybutyrylation Bridging Energy Metabolism With Transcription and Protein Functional Remodeling. Nutr. Rev..

[31] Rivera C., Gurard-Levin Z.A., Almouzni G., Loyola A. (2014). Histone Lysine Methylation and Chromatin Replication. Biochim. Biophys. Acta.

[32] Zhou T., Cheng X., He Y., Xie Y., Xu F., Xu Y., Huang W. (2022). Function and Mechanism of Histone β-Hydroxybutyrylation in Health and Disease. Front. Immunol..

[33] Ehtiati S., Hatami B., Khatami S.H., Tajernarenj K., Abdi S., Sirati-Sabet M., Ghazizadeh Hashemi S.A.H., Ahmadzade R., Hamed N., Goudarzi M. (2025). The Multifaceted Influence of Beta-Hydroxybutyrate on Autophagy, Mitochondrial Metabolism, and Epigenetic Regulation. J. Cell Biochem..

[34] Zhang D., Tang Z., Huang H., Zhou G., Cui C., Weng Y., Liu W., Kim S., Lee S., Perez-Neut M. (2019). Metabolic Regulation of Gene Expression by Histone Lactylation. Nature.

[35] Juge N., Gray J.A., Omote H., Miyaji T., Inoue T., Hara C., Uneyama H., Edwards R.H., Nicoll R.A., Moriyama Y. (2010). Metabolic Control of Vesicular Glutamate Transport and Release. Neuron.

[36] Hone-Blanchet A., Antal B., McMahon L., Lithen A., Smith N.A., Stufflebeam S., Yen Y.-F., Lin A., Jenkins B.G., Mujica-Parodi L.R. (2023). Acute Administration of Ketone Beta-Hydroxybutyrate Downregulates 7T Proton Magnetic Resonance Spectroscopy-Derived Levels of Anterior and Posterior Cingulate GABA and Glutamate in Healthy Adults. Neuropsychopharmacology.

[37] Van Nieuwenhuizen H., Antal B.B., Hone-Blanchet A., Lithen A., McMahon L., Nikolaidou S., Kuang Z., Clarke K., Jenkins B.G., Rothman D.L. (2025). Ketosis Elevates Antioxidants and Markers of Energy Metabolism: A Proton Magnetic Resonance Spectroscopy Study. Biol. Psychiatry Cogn. Neurosci. Neuroimaging.

[38] Rahman M., Muhammad S., Khan M.A., Chen H., Ridder D.A., Müller-Fielitz H., Pokorná B., Vollbrandt T., Stölting I., Nadrowitz R. (2014). The β-Hydroxybutyrate Receptor HCA2 Activates a Neuroprotective Subset of Macrophages. Nat. Commun..

[39] Youm Y.-H., Nguyen K.Y., Grant R.W., Goldberg E.L., Bodogai M., Kim D., D’Agostino D., Planavsky N., Lupfer C., Kanneganti T.D. (2015). The Ketone Metabolite β-Hydroxybutyrate Blocks NLRP3 Inflammasome–Mediated Inflammatory Disease. Nat. Med..

[40] Abdelrahman A.A., Powell F.L., Jadeja R.N., Jones M.A., Thounaojam M.C., Bartoli M., Al-Shabrawey M., Martin P.M. (2022). Expression and Activation of the Ketone Body Receptor HCAR2/GPR109A Promotes Preservation of Retinal Endothelial Cell Barrier Function. Exp. Eye Res..

[41] Leonard W.R., Robertson M.L. (1992). Nutritional Requirements and Human Evolution: A Bioenergetics Model. Am. J. Hum. Biol..

[42] Fonseca-Azevedo K., Herculano-Houzel S. (2012). Metabolic Constraint Imposes Tradeoff between Body Size and Number of Brain Neurons in Human Evolution. Proc. Natl. Acad. Sci. USA.

[43] Leigh S.R. (2004). Brain Growth, Life History, and Cognition in Primate and Human Evolution. Am. J. Primatol..

[44] Bier D.M., Leake R.D., Haymond M.W., Arnold K.J., Gruenke L.D., Sperling M.A., Kipnis D.M. (1977). Measurement of “True” Glucose Production Rates in Infancy and Childhood with 6,6-Dideuteroglucose. Diabetes.

[45] Bougnères P.F., Lemmel C., Ferré P., Bier D.M. (1986). Ketone Body Transport in the Human Neonate and Infant. J. Clin. Investig..

[46] Widdowson E.M. (1950). Chemical Composition of Newly Born Mammals. Nature.

[47] Bougnères P.F., Karl I.E., Hillman L.S., Bier D.M. (1982). Lipid Transport in the Human Newborn. J. Clin. Investig..

[48] Balasse E.O. (1979). Kinetics of Ketone Body Metabolism in Fasting Humans. Metabolism.

[49] Owen O.E., Felig P., Morgan A.P., Wahren J., Cahill G.F. (1969). Liver and Kidney Metabolism during Prolonged Starvation. J. Clin. Investig..

[50] Garber A.J., Menzel P.H., Boden G., Owen O.E. (1974). Hepatic Ketogenesis and Gluconeogenesis in Humans. J. Clin. Investig..

[51] Owen O.E., Caprio S., Reichard G.A., Mozzoli M.A., Boden G., Owen R.S. (1983). Ketosis of Starvation: A Revisit and New Perspectives. Clin. Endocrinol. Metab..

[52] Balasse E.O., Féry F. (1989). Ketone Body Production and Disposal: Effects of Fasting, Diabetes, and Exercise. Diabetes Metab. Rev..

[53] Bougnères P.F., Ferre P. (1987). Study of Ketone Body Kinetics in Children by a Combined Perfusion of 13C and 2H3 Tracers. Am. J. Physiol. Endocrinol. Metab..

[54] Settergren G., Lindblad B.S., Persson B. (1976). Cerebral Blood Flow and Exchange of Oxygen, Glucose, Ketone Bodies, Lactate, Pyruvate and Amino Acids in Infants. Acta Paediatr..

[55] Kuzawa C.W., Chugani H.T., Grossman L.I., Lipovich L., Muzik O., Hof P.R., Wildman D.E., Sherwood C.C., Leonard W.R., Lange N. (2014). Metabolic Costs and Evolutionary Implications of Human Brain Development. Proc. Natl. Acad. Sci. USA.

[56] Settergren G., Lindblad B.S., Persson B. (1980). Cerebral Blood Flow and Exchange of Oxygen, Glucose Ketone Bodies, Lactate, Pyruvate and Amino Acids in Anesthetized Children. Acta Paediatr..

[57] Saudubray J.M., Marsac C., Limal J.M., Dumurgier E., Charpentier C., Ogier H., Coudè F.X. (1981). Variation in Plasma Ketone Bodies during a 24-Hour Fast in Normal and in Hypoglycemic Children: Relationship to Age. J. Pediatr..

[58] Haymond M.W., Karl I.E., Clarke W.L., Pagliara A.S., Santiago J.V. (1982). Differences in Circulating Gluconeogenic Substrates during Short-Term Fasting in Men, Women, and Children. Metabolism.

[59] Owen O.E., Morgan A.P., Kemp H.G., Sullivan J.M., Herrera M.G., Cahill G.F. (1967). Brain Metabolism during Fasting. J. Clin. Investig..

[60] Holliday M.A., Falkner F., Tanner J.M. (1978). Body Composition and Energy Needs during Growth. Human Growth.

[61] Goyal M.S., Iannotti L.L., Raichle M.E. (2018). Brain Nutrition: A Life Span Approach. Annu. Rev. Nutr..

[62] Miller D.J., Duka T., Stimpson C.D., Schapiro S.J., Baze W.B., McArthur M.J., Fobbs A.J., Sousa A.M.M., Šestan N., Wildman D.E. (2012). Prolonged Myelination in Human Neocortical Evolution. Proc. Natl. Acad. Sci. USA.

[63] Petanjek Z., Judaš M., Šimić G., Rašin M.R., Uylings H.B.M., Rakic P., Kostović I. (2011). Extraordinary Neoteny of Synaptic Spines in the Human Prefrontal Cortex. Proc. Natl. Acad. Sci. USA.

[64] Fayol L., Baud O., Monier A., Pellerin L., Magistretti P., Evrard P., Verney C. (2004). Immunocytochemical Expression of Monocarboxylate Transporters in the Human Visual Cortex at Midgestation. Brain Res. Dev. Brain Res..

[65] Baud O., Fayol L., Gressens P., Pellerin L., Magistretti P., Evrard P., Verney C. (2003). Perinatal and Early Postnatal Changes in the Expression of Monocarboxylate Transporters MCT1 and MCT2 in the Rat Forebrain. J. Comp. Neurol..

[66] Adam P.A.J., Räihä N., Rähialä E., Kekomäki M. (1975). Oxidation of Glucose and D-B-OH-Butyrate by the Early Human Fetal Brain. Acta Paediatr..

[67] Page M.A., Williamson D.H. (1971). Enzymes of Ketone-Body Utilisation in Human Brain. Lancet.

[68] Patel M.S., Johnson C.A., Rajan R., Owen O.E. (1975). The Metabolism of Ketone Bodies in Developing Human Brain: Development of Ketone-body-utilizing Enzymes and Ketone Bodies as Precursors for Lipid Synthesis. J. Neurochem..

[69] Page M.A., Krebs H.A., Williamson D.H. (1971). Activities of Enzymes of Ketone-Body Utilization in Brain and Other Tissues of Suckling Rats. Biochem. J..

[70] Lockwood E.A., Bailey E. (1971). The Course of Ketosis and the Activity of Key Enzymes of Ketogenesis and Ketone-Body Utilization during Development of the Postnatal Rat. Biochem. J..

[71] Hawkins R.A., Williamson D.H., Krebs H.A. (1971). Ketone-Body Utilization by Adult and Suckling Rat Brain in Vivo. Biochem. J..

[72] Koper J.W., Lopes-Cardozo M., Van Golde L.M.G. (1981). Preferential Utilization of Ketone Bodies for the Synthesis of Myelin Cholesterol in Vivo. Biochim. Biophys. Acta.

[73] Kaye R., Davidson M.H., Williams M.L., Kumagai M., Picou D.M. (1961). The Response of Blood Glucose, Ketones, and Plasma Nonesterified Fatty Acids to Fasting and Epinephrine Injection in Infants and Children. J. Pediatr..

[74] Krainick H.G., Russel W. (1958). Klinische Und Experimentelle Beobachtungen Zur Acetonämie Im Kindesalter. Modern Problems in Pediatrics.

[75] Kleinbaum H., Weinke I. (1967). Nüchternwerte und 24-Std-Rhythmik des Ketonkörpergehaltes im Blut gesunder Säuglinge verschiedener Altersstufen. Z. Kinderheilkd..

[76] Owen O.E. (2005). Ketone Bodies as a Fuel for the Brain during Starvation. Biochem. Mol. Bio Educ..

[77] Gottstein U., Müller W., Berghoff W., Gärtner H., Held K. (1971). Zur Utilisation von nicht-veresterten Fettsäuren und Ketonkörpern im Gehirn des Menschen. Klin. Wochenschr..

[78] Lying-Tunell U., Lindblad B.S., Malmlund H.O., Persson B. (2009). Cerebral Blood Flow and Metabolic Rate of Oxygen, Glucose, Lactate, Pyruvate, Ketone Bodies and Amino Acids: I. YOUNG AND OLD NORMAL SUBJECTS. Acta Neurol. Scand..

[79] Hasselbalch S.G., Knudsen G.M., Jakobsen J., Hageman L.P., Holm S., Paulson O.B. (1994). Brain Metabolism during Short-Term Starvation in Humans. J. Cereb. Blood Flow. Metab..

[80] Blomqvist G., Thorell J.O., Ingvar M., Widen L., Stone-Elander S. (1995). Use of R-Beta-[1-11C]Hydroxybutyrate in PET Studies of Regional Cerebral Uptake of Ketone Bodies in Humans. Am. J. Physiol. Endocrinol. Metab..

[81] Blomqvist G., Alvarsson M., Grill V., Von Heijne G., Ingvar M., Thorell J.O., Stone-Elander S., Widén L., Ekberg K. (2002). Effect of Acute Hyperketonemia on the Cerebral Uptake of Ketone Bodies in Nondiabetic Subjects and IDDM Patients. Am. J. Physiol. Endocrinol. Metab..

[82] Courchesne-Loyer A., Croteau E., Castellano C.-A., St-Pierre V., Hennebelle M., Cunnane S.C. (2017). Inverse Relationship between Brain Glucose and Ketone Metabolism in Adults during Short-Term Moderate Dietary Ketosis: A Dual Tracer Quantitative Positron Emission Tomography Study. J. Cereb. Blood Flow. Metab..

[83] Zhu H., Barker P.B., Modo M., Bulte J.W.M. (2011). MR Spectroscopy and Spectroscopic Imaging of the Brain. Magnetic Resonance Neuroimaging.

[84] Pifferi F., Tremblay S., Croteau E., Fortier M., Tremblay-Mercier J., Lecomte R., Cunnane S.C. (2011). Mild Experimental Ketosis Increases Brain Uptake of 11C-Acetoacetate and 18F-Fluorodeoxyglucose: A Dual-Tracer PET Imaging Study in Rats. Nutr. Neurosci..

[85] Leino R.L., Gerhart D.Z., Duelli R., Enerson B.E., Drewes L.R. (2001). Diet-Induced Ketosis Increases Monocarboxylate Transporter (MCT1) Levels in Rat Brain. Neurochem. Int..

[86] Johnson R.H., Walton J.L., Krebs H.A., Williamson D.H. (1969). Post-Exercise Ketosis. Lancet.

[87] Evans M., Cogan K.E., Egan B. (2017). Metabolism of Ketone Bodies during Exercise and Training: Physiological Basis for Exogenous Supplementation. J. Physiol..

[88] Fery F., Balasse E.O. (1983). Ketone Body Turnover during and after Exercise in Overnight-Fasted and Starved Humans. Am. J. Physiol. Endocrinol. Metab..

[89] Butte N.F., Wong W.W., Treuth M.S., Ellis K.J., O’Brian Smith E. (2004). Energy Requirements during Pregnancy Based on Total Energy Expenditure and Energy Deposition. Am. J. Clin. Nutr..

[90] Phelps R.L., Metzger B.E., Freinkel N. (1981). Carbohydrate Metabolism in Pregnancy: XVII. Diurnal Profiles of Plasma Glucose, Insulin, Free Fatty Acids, Triglycerides, Cholesterol, and Individual Amino Acids in Late Normal Pregnancy. Am. J. Obstet. Gynecol..

[91] Felig P., Lynch V. (1970). Starvation in Human Pregnancy: Hypoglycemia, Hypoinsulinemia, and Hyperketonemia. Science.

[92] Bon C., Golfier F., Poloce F., Champion F., Pichot J., Revol A. (2007). Métabolisme Fœto-Maternel Au Cours de Grossesses Humaines Normales: Étude de 73 Cas. Ann. Biol. Clin..

[93] Kim Y.J., Lynch V., Felig P., Cook C.D. (1971). Fetal-Maternal Metabolic Fuel Adaptations to Caloric Deprivation in Human Pregnancy. Pediatr. Res..

[94] Mohammad M.A., Sunehag A.L., Chacko S.K., Pontius A.S., Maningat P.D., Haymond M.W. (2009). Mechanisms to Conserve Glucose in Lactating Women during a 42-h Fast. Am. J. Physiol. Endocrinol. Metab..

[95] Szili-Torok T., De Borst M.H., Garcia E., Gansevoort R.T., Dullaart R.P.F., Connelly M.A., Bakker S.J.L., Tietge U.J.F. (2023). Fasting Ketone Bodies and Incident Type 2 Diabetes in the General Population. Diabetes.

[96] Knol M.G.E., Van Der Vaart A., Kieneker L., Connelly M.A., Bakker S.J.L., Müller R.-U., Rinschen M.M., Gansevoort R.T., Van Gastel M.D.A. (2025). Sex-Specific Determinants of the Ketone Body β-Hydroxybutyrate in the General Population. J. Clin. Endocrinol. Metab..

[97] London E.D., Margolin R.A., Duara R., Holloway H.W., Robertson-tchabo E.A., Cutler N.R., Rapoport S.I. (1986). Effects of Fasting on Ketone Body Concentrations in Healthy Men of Different Ages. J. Gerontol..

[98] Freemantle E., Vandal M., Tremblay-Mercier J., Plourde M., Poirier J., Cunnane S.C. (2009). Metabolic Response to a Ketogenic Breakfast in the Healthy Elderly. J. Nutr. Health Aging.

[99] Nugent S., Tremblay S., Chen K.W., Ayutyanont N., Roontiva A., Castellano C.-A., Fortier M., Roy M., Courchesne-Loyer A., Bocti C. (2014). Brain Glucose and Acetoacetate Metabolism: A Comparison of Young and Older Adults. Neurobiol. Aging.

[100] Thayer Z.M., Rutherford J., Kuzawa C.W. (2020). The Maternal Nutritional Buffering Model: An Evolutionary Framework for Pregnancy Nutritional Intervention. Evol. Med. Public. Health.

[101] Bateson P., Barker D., Clutton-Brock T., Deb D., D’Udine B., Foley R.A., Gluckman P., Godfrey K., Kirkwood T., Lahr M.M. (2004). Developmental Plasticity and Human Health. Nature.

[102] Horton T.H. (2005). Fetal Origins of Developmental Plasticity: Animal Models of Induced Life History Variation. Am. J. Hum. Biol..

[103] Kuzawa C.W. (2005). Fetal Origins of Developmental Plasticity: Are Fetal Cues Reliable Predictors of Future Nutritional Environments?. Am. J. Hum. Biol..

[104] Jablonka E., Lamb M.J. (1995). Epigenetic Inheritance and Evolution: The Lamarckian Dimension.

[105] Kuzawa C.W. (2017). Which Environments Matter in Studies of Early Life Developmental Plasticity?. Evol. Med. Public. Health.

[106] Lea A.J., Tung J., Archie E.A., Alberts S.C. (2017). Developmental Plasticity: Bridging Research in Evolution and Human Health. Evol. Med. Public. Health.

[107] Foley R.A., Lee P.C. (1991). Ecology and Energetics of Encephalization in Hominid Evolution. Philos. Trans. R. Soc. Lond. B Biol. Sci..

[108] Gluckman P.D., Hanson M.A., Bateson P., Beedle A.S., Law C.M., Bhutta Z.A., Anokhin K.V., Bougnères P., Chandak G.R., Dasgupta P. (2009). Towards a New Developmental Synthesis: Adaptive Developmental Plasticity and Human Disease. Lancet.

[109] DeSilva J.M., Lesnik J.J. (2008). Brain Size at Birth throughout Human Evolution: A New Method for Estimating Neonatal Brain Size in Hominins. J. Hum. Evol..

[110] Beja-Pereira A., Luikart G., England P.R., Bradley D.G., Jann O.C., Bertorelle G., Chamberlain A.T., Nunes T.P., Metodiev S., Ferrand N. (2003). Gene-Culture Coevolution between Cattle Milk Protein Genes and Human Lactase Genes. Nat. Genet..

[111] Kuzawa C.W. (1998). Adipose Tissue in Human Infancy and Childhood: An Evolutionary Perspective. Am. J. Phys. Anthropol..

[112] Friedemann T.E. (1928). Starvation Ketosis of the Primates. Science.

[113] Levitsky L.L., Fisher D.E., Paton J.B., Delannoy C.W. (1977). Fasting Plasma Levels of Glucose, Acetoacetate, D-β-Hydroxybutyrate, Glycerol, and Lactate in the Baboon Infant: Correlation with Cerebral Uptake of Substrates and Oxygen. Pediatr. Res..

[114] Girard J., Ferre P., Pegorier J.P., Duee P.H. (1992). Adaptations of Glucose and Fatty Acid Metabolism during Perinatal Period and Suckling-Weaning Transition. Physiol. Rev..

[115] Singh R.B. (2013). Nutrition in Transition from Homo Sapiens to Homo Economicus. Open Nutraceuticals J..

[116] Kelly R.L. (2013). The Lifeways of Hunter-Gatherers: The Foraging Spectrum.

[117] Cordain L., Miller J.B., Eaton S.B., Mann N., Holt S.H., Speth J.D. (2000). Plant-Animal Subsistence Ratios and Macronutrient Energy Estimations in Worldwide Hunter-Gatherer Diets. Am. J. Clin. Nutr..

[118] Veile A. (2018). Hunter-Gatherer Diets and Human Behavioral Evolution. Physiol. Behav..

[119] Marlowe F.W. (2005). Hunter-Gatherers and Human Evolution. Evol. Anthropol..

[120] Hinde K., German J.B. (2012). Food in an Evolutionary Context: Insights from Mother’s Milk. J. Sci. Food Agric..

[121] Prentice A.M., Goldberg G.R. (2000). Energy Adaptations in Human Pregnancy: Limits and Long-Term Consequences. Am. J. Clin. Nutr..

[122] Chakravarthy M.V., Booth F.W. (2004). Eating, Exercise, and “Thrifty” Genotypes: Connecting the Dots toward an Evolutionary Understanding of Modern Chronic Diseases. J. Appl. Physiol..

[123] Freese J., Klement R.J., Ruiz-Núñez B., Schwarz S., Lötzerich H. (2018). The Sedentary (r)Evolution: Have We Lost Our Metabolic Flexibility?. F1000Res.

[124] Shambaugh G.E., Mrozak S.C., Freinkel N. (1977). Fetal Fuels. I. Utilization of Ketones by Isolated Tissues at Various Stages of Maturation and Maternal Nutrition during Late Gestation. Metabolism.

[125] Dahlquist G., Persson B. (1976). The Rate of Cerebral Utilization of Glucose, Ketone Bodies, and Oxygen: A Comparative in Vivo Study of Infant and Adult Rats. Pediatr. Res..

[126] Klee C.B., Sokoloff L. (1967). Changes in d(-)-β-Hydroxybutyric Dehydrogenase Activity during Brain Maturation in the Rat. J. Biol. Chem..

[127] Middleton B. (1973). The Acetoacetyl-Coenzyme A Thiolases of Rat Brain and Their Relative Activities during Postnatal Development. Biochem. J..

[128] Cremer J.E., Braun L.D., Oldendorf W.H. (1976). Changes during Development in Transport Processes of the Blood-Brain Barrier. Biochim. Biophys. Acta.

[129] Rafiki A., Boulland J.L., Halestrap A.P., Ottersen O.P., Bergersen L. (2003). Highly Differential Expression of the Monocarboxylate Transporters MCT2 and MCT4 in the Developing Rat Brain. Neuroscience.

[130] Cremer J.E., Heath D.F. (1974). The Estimation of Rates of Utilization of Glucose and Ketone Bodies in the Brain of the Suckling Rat Using Compartmental Analysis of Isotopic Data. Biochem. J..

[131] Ruderman N.B., Ross P.S., Berger M., Goodman M.N. (1974). Regulation of Glucose and Ketone-Body Metabolism in Brain of Anaesthetized Rats. Biochem. J..

[132] Jiang L., Mason G.F., Rothman D.L., De Graaf R.A., Behar K.L. (2011). Cortical Substrate Oxidation during Hyperketonemia in the Fasted Anesthetized Rat in Vivo. J. Cereb. Blood Flow. Metab..

[133] Bentourkia M., Tremblay S., Pifferi F., Rousseau J., Lecomte R., Cunnane S. (2009). PET Study of 11C-Acetoacetate Kinetics in Rat Brain during Dietary Treatments Affecting Ketosis. Am. J. Physiol. Endocrinol. Metab..

[134] Roy M., Nugent S., Tremblay S., Descoteaux M., Beaudoin J.-F., Tremblay L., Lecomte R., Cunnane S.C. (2013). A Dual Tracer PET-MRI Protocol for the Quantitative Measure of Regional Brain Energy Substrates Uptake in the Rat. J. Vis. Exp..

[135] Cremer J.E. (1980). Measurement of Brain Substrate Utilization in Adult and Infant Rats Using Various l4C-Labeled Precursors. Cerebral Metabolism and Neural Function.

[136] LaManna J.C., Salem N., Puchowicz M., Erokwu B., Koppaka S., Flask C., Lee Z., Liss P., Hansell P., Bruley D.F., Harrison D.K. (2009). Ketones Suppress Brain Glucose Consumption. Oxygen Transport to Tissue XXX.

[137] Zhang Y., Kuang Y., Xu K., Harris D., Lee Z., LaManna J., Puchowicz M.A. (2013). Ketosis Proportionately Spares Glucose Utilization in Brain. J. Cereb. Blood Flow. Metab..

[138] Girard J.R., Ferré P., Gilbert M., Kervran A., Assan R., Marliss E.B. (1977). Fetal Metabolic Response to Maternal Fasting in the Rat. Am. J. Physiol. Endocrinol. Metab..

[139] Herrera E. (2000). Metabolic Adaptations in Pregnancy and Their Implications for the Availability of Substrates to the Fetus. Eur. J. Clin. Nutr..

[140] Herrera E., Freinkel N. (1969). Carbohydrate Metabolism in Pregnancy. J. Clin. Investig..

[141] Scow R.O., Chernick S.S., Brinley M.S. (1964). Hyperlipemia and Ketosis in the Pregnant Rat. Am. J. Physiol..

[142] Holcomb L.E., O’Neill C.C., DeWitt E.A., Kolwicz S.C. (2021). The Effects of Fasting or Ketogenic Diet on Endurance Exercise Performance and Metabolism in Female Mice. Metabolites.

[143] Padilla C.J., Harris H., Volek J.S., Clark B.C., Arnold W.D. (2023). Ketogenic Diet Improves Motor Function and Motor Unit Connectivity in Aged C57BL/6 Mice. Res. Sq..

[144] Takahashi S. (2020). Metabolic Compartmentalization between Astroglia and Neurons in Physiological and Pathophysiological Conditions of the Neurovascular Unit. Neuropathology.

[145] Shalamu A., Dong Z., Liu B., Pan L., Cai Y., Liu L., Ma X., Hu K., Sun A., Ge J. (2022). Effects of the Ketogenic Diet in Mice with Hind Limb Ischemia. Nutr. Metab..

[146] Ma S., Wu C., Tong Y., Takahashi Y., Suzuki K., Hara T. (2025). Ketone Body Supplementation in Keto-adapted Mice Reveals Metabolic Adaptations and Glycogen-independent Exercise Capacity. Physiol. Rep..

[147] Fu J., Liu S., Li M., Guo F., Wu X., Hu J., Wen L., Wang J., Li X. (2024). Optimal Fasting Duration for Mice as Assessed by Metabolic Status. Sci. Rep..

[148] Sunny N.E., Satapati S., Fu X., He T., Mehdibeigi R., Spring-Robinson C., Duarte J., Potthoff M.J., Browning J.D., Burgess S.C. (2010). Progressive Adaptation of Hepatic Ketogenesis in Mice Fed a High-Fat Diet. Am. J. Physiol. Endocrinol. Metab..

[149] Newman J.C., Covarrubias A.J., Zhao M., Yu X., Gut P., Ng C.-P., Huang Y., Haldar S., Verdin E. (2017). Ketogenic Diet Reduces Midlife Mortality and Improves Memory in Aging Mice. Cell Metab..

[150] Wang L., Xing X., Zeng X., Jackson S.R., TeSlaa T., Al-Dalahmah O., Samarah L.Z., Goodwin K., Yang L., McReynolds M.R. (2022). Spatially Resolved Isotope Tracing Reveals Tissue Metabolic Activity. Nat. Methods.

[151] Andrews M.T. (2019). Molecular Interactions Underpinning the Phenotype of Hibernation in Mammals. J. Exp. Biol..

[152] Schwartz C., Hampton M., Andrews M.T. (2013). Seasonal and Regional Differences in Gene Expression in the Brain of a Hibernating Mammal. PLoS ONE.

[153] Epperson L.E., Karimpour-Fard A., Hunter L.E., Martin S.L. (2011). Metabolic Cycles in a Circannual Hibernator. Physiol. Genom..

[154] Russeth K.P., Higgins L., Andrews M.T. (2006). Identification of Proteins from Non-Model Organisms Using Mass Spectrometry: Application to a Hibernating Mammal. J. Proteome Res..

[155] David Lust W., Wheaton A.B., Feussner G., Passonneau J. (1989). Metabolism in the Hamster Brain during Hibernation and Arousal. Brain Res..

[156] Mountassif D., Kabine M., Mounchid K., Mounaji K., Latruffe N., El Kebbaj M.S. (2009). Sensitivity of Liver Metabolism in Jerboa (Jaculus Orientalis) to Ciprofibrate, a Peroxisome Proliferator. Mol. Med. Rep..

[157] Henry P., Russeth K.P., Tkac I., Drewes L.R., Andrews M.T., Gruetter R. (2007). Brain Energy Metabolism and Neurotransmission at Near-freezing Temperatures: In Vivo^1^ H MRS Study of a Hibernating Mammal. J. Neurochem..

[158] Shi Z., Wang X., Duan W., Du Y., Ling S., Zhang Z., Wang G., Zhao D., Ding J., Zhang K. (2026). Neuroplasticity and Brain Health: Insights from Natural Torpor. Biol. Rev. Camb. Philos. Soc..

[159] Longo V.D., Mattson M.P. (2014). Fasting: Molecular Mechanisms and Clinical Applications. Cell Metab..

[160] Lee J.H., Verma N., Yeung C., Sung H.-K. (2020). Intermittent Fasting: Physiological Implications on Outcomes in Mice and Men. Physiology.

[161] Wilkinson J.F. (1997). Look at Me. Smithson. Mag..

[162] Conklin H.W. (1922). Cause and Treatment of Epilepsy. J. Am. Osteopath..

[163] Lennox W.G. (1928). Studies in Epilepsy. VIII: The Clinical Effect of Fasting. Arch. Neur. Psych..

[164] Higgins H.L. (1930). Some Physiological and Clinical Effects of High Fat Feeding. N. Engl. J. Med..

[165] Geyelin H.R. (1921). Fasting as a Method for Treating Epilepsy. Med. Rec..

[166] Geyelin H.R. (1929). The Relation of Chemical Influences, Including Diet and Endocrine Disturbances, to Epilepsy. Ann. Intern. Med..

[167] Kennaway E.L. (1914). The Relative Amounts of β-Hydroxybutric Acid and Aceto-Acetic Acid Excreted in Acetonuria. Biochem. J..

[168] Woodyatt R.T. (1921). Objects and Method of Diet Adjustment in Diabetics. Arch. Intern. Med..

[169] Wilder R.M. (1921). The Effect on Ketonemia on the Course of Epilepsy. Mayo Clin. Bull..

[170] Wilder R.M. (1921). High Fat Diets in Epilepsy. Mayo Clin. Bull..

[171] Peterman M.G. (1924). The Ketogenic Diet in the Treatment of Epilepsy: A Preliminary Report. Am. J. Dis. Child..

[172] Talbot F.B., Metcalf K., Moriarty M.E. (1926). The Ketogenic Diet in the Treatment of Idiopathic Epilepsy. Am. J. Dis. Child..

[173] Talbot F.B., Metcalf K.M., Moriarty M.E. (1926). A Clinical Study of Epileptic Children Treated by the Ketogenic Diet. Boston Med. Surg. J..

[174] Talbot F.B., Metcalf K.M., Moriarty M.E. (1927). Epilepsy: Chemical Investigations of Rational Treatment by Production of Ketosis. Am. J. Dis. Child..

[175] Talbot F.B. (1927). The Treatment of Epilepsy of Childhood by the Ketogenic Diet. R. I Med. J..

[176] Barborka C.J. (1928). Ketogenic Diet Treatment of Epilepsy in Adults. JAMA.

[177] Helmholz H.F. (1927). The Treatment of Epilepsy in Childhood—Five Year’s Experience with the Ketogenic Diet. JAMA.

[178] Barborka C.J. (1930). Epilepsy in Adults: Results of Treatment by Ketogenic Diet in One Hundred Cases. Arch. Neur. Psych..

[179] Ford F. (1937). The Epilepsies and Paroxysmal Disorders of the Nervous System.

[180] Ballard O., Morrow A.L. (2013). Human Milk Composition: Nutrients and Bioactive Factors. Pediatr. Clin. N. Am..

[181] Hellerstein M.K., Neese R.A., Linfoot P., Christiansen M., Turner S., Letscher A. (1997). Hepatic Gluconeogenic Fluxes and Glycogen Turnover during Fasting in Humans. A Stable Isotope Study. J. Clin. Investig..

[182] Bougnères P.-F., Saudubray J.-M., Marsac C., Bernard O., Odièvre M., Girard J. (1981). Fasting Hypoglycemia Resulting from Hepatic Carnitine Palmitoyl Transferase Deficiency. J. Pediatr..

[183] Thevenet J., De Marchi U., Domingo J.S., Christinat N., Bultot L., Lefebvre G., Sakamoto K., Descombes P., Masoodi M., Wiederkehr A. (2016). Medium-chain Fatty Acids Inhibit Mitochondrial Metabolism in Astrocytes Promoting Astrocyte-neuron Lactate and Ketone Body Shuttle Systems. FASEB J..

[184] Courchesne-Loyer A., Fortier M., Tremblay-Mercier J., Chouinard-Watkins R., Roy M., Nugent S., Castellano C.-A., Cunnane S.C. (2013). Stimulation of Mild, Sustained Ketonemia by Medium-Chain Triacylglycerols in Healthy Humans: Estimated Potential Contribution to Brain Energy Metabolism. Nutrition.

[185] Nonaka H., Ohue-Kitano R., Masujima Y., Igarashi M., Kimura I. (2022). Dietary Medium-Chain Triglyceride Decanoate Affects Glucose Homeostasis Through GPR84-Mediated GLP-1 Secretion in Mice. Front. Nutr..

[186] Shobako M., Kawano K., Taniguchi E., Ohinata K. (2025). Medium-Chain Triglycerides Tricaprin TC10 and Tricaprylin TC8 Attenuated HFD-Induced Cognitive Decline in a Manner Dependent on or Independent of GLP-1. Sci. Rep..

[187] Li Y., Perry T., Kindy M.S., Harvey B.K., Tweedie D., Holloway H.W., Powers K., Shen H., Egan J.M., Sambamurti K. (2009). GLP-1 Receptor Stimulation Preserves Primary Cortical and Dopaminergic Neurons in Cellular and Rodent Models of Stroke and Parkinsonism. Proc. Natl. Acad. Sci. USA.

[188] Hashim S.A., VanItallie T.B. (2014). Ketone Body Therapy: From the Ketogenic Diet to the Oral Administration of Ketone Ester. J. Lipid Res..

[189] Bohnen J.L.B., Albin R.L., Bohnen N.I. (2023). Ketogenic Interventions in Mild Cognitive Impairment, Alzheimer’s Disease, and Parkinson’s Disease: A Systematic Review and Critical Appraisal. Front. Neurol..

[190] Jensen N.J., Wodschow H.Z., Nilsson M., Rungby J. (2020). Effects of Ketone Bodies on Brain Metabolism and Function in Neurodegenerative Diseases. Int. J. Mol. Sci..

[191] Pawlosky R.J., Kashiwaya Y., King M.T., Veech R.L. (2020). A Dietary Ketone Ester Normalizes Abnormal Behavior in a Mouse Model of Alzheimer’s Disease. Int. J. Mol. Sci..

[192] Chen O., Blonquist T., Mah E., Sanoshy K., Beckman D., Nieman K., Winters B., Anthony J., Verdin E., Newman J. (2021). Tolerability and Safety of a Novel Ketogenic Ester, Bis-Hexanoyl (R)-1,3-Butanediol: A Randomized Controlled Trial in Healthy Adults. Nutrients.

[193] Crabtree C.D., Blade T., Hyde P.N., Buga A., Kackley M.L., Sapper T.N., Panda O., Roa-Diaz S., Anthony J.C., Newman J.C. (2023). Bis Hexanoyl (R)-1,3-Butanediol, a Novel Ketogenic Ester, Acutely Increases Circulating r- and s-ß-Hydroxybutyrate Concentrations in Healthy Adults. Am. J. Clin. Nutr..

[194] Poff A.M., D’Agostino D.P. (2019). Ketone Administration for Seizure Disorders: History and Rationale for Ketone Esters and Metabolic Alternatives. Front. Neurosci..

[195] Soliven M.A., Rogers C.Q., Williams M.S., Thomas N.N., Turos E., D’Agostino D.P. (2024). Oral Administration of a Novel, Synthetic Ketogenic Compound Elevates Blood β-Hydroxybutyrate Levels in Mice in Both Fasted and Fed Conditions. Nutrients.

[196] Avgerinos K.I., Mullins R.J., Egan J.M., Kapogiannis D. (2022). Ketone Ester Effects on Biomarkers of Brain Metabolism and Cognitive Performance in Cognitively Intact Adults ≥ 55 Years Old. A Study Protocol for a Double-Blinded Randomized Controlled Clinical Trial. J. Prev. Alzheimers Dis..

[197] Wang Y., Zhang J., Zhang Y., Yao J. (2023). Bibliometric Analysis of Global Research Profile on Ketogenic Diet Therapies in Neurological Diseases: Beneficial Diet Therapies Deserve More Attention. Front. Endocrinol..

[198] Ruan Y., Chen L., She D., Chung Y., Ge L., Han L. (2022). Ketogenic Diet for Epilepsy: An Overview of Systematic Review and Meta-Analysis. Eur. J. Clin. Nutr..

[199] Lyons L., Schoeler N.E., Langan D., Cross J.H. (2020). Use of Ketogenic Diet Therapy in Infants with Epilepsy: A Systematic Review and Meta-analysis. Epilepsia.

[200] Prasad A., Glover C., Shuler M.S., Dayal V., Lithander F.E. (2025). The Effect of Diet on Parkinson’s Disease Progression, Symptoms and Severity: A Review of Randomised Controlled Trials. Proc. Nutr. Soc..

[201] Choi A., Hallett M., Ehrlich D. (2021). Nutritional Ketosis in Parkinson’s Disease—A Review of Remaining Questions and Insights. Neurotherapeutics.

[202] Brockhoff J.D., Bereswill S., Heimesaat M.M. (2023). The Impact of Ketogenic Diet on the Onset and Progression of Multiple Sclerosis. Eur. J. Microbiol. Immunol..

[203] Neri L.D.C.L., Ferraris C., Catalano G., Guglielmetti M., Pasca L., Pezzotti E., Carpani A., Tagliabue A. (2023). Ketosis and Migraine: A Systematic Review of the Literature and Meta-Analysis. Front. Nutr..

[204] Arora N., Litofsky N.S., Golzy M., Aneja R., Staudenmyer D., Qualls K., Patil S. (2022). Phase I Single Center Trial of Ketogenic Diet for Adults with Traumatic Brain Injury. Clin. Nutr. ESPEN.

[205] Har-Even M., Rubovitch V., Ratliff W.A., Richmond-Hacham B., Citron B.A., Pick C.G. (2021). Ketogenic Diet as a Potential Treatment for Traumatic Brain Injury in Mice. Sci. Rep..

[206] Jang J., Kim S.R., Lee J.E., Lee S., Son H.J., Choe W., Yoon K.-S., Kim S.S., Yeo E.-J., Kang I. (2023). Molecular Mechanisms of Neuroprotection by Ketone Bodies and Ketogenic Diet in Cerebral Ischemia and Neurodegenerative Diseases. Int. J. Mol. Sci..

[207] Bostock E.C.S., Kirkby K.C., Taylor B.V.M. (2017). The Current Status of the Ketogenic Diet in Psychiatry. Front. Psychiatry.

[208] Choi J., Kang J., Kim T., Nehs C.J. (2024). Sleep, Mood Disorders, and the Ketogenic Diet: Potential Therapeutic Targets for Bipolar Disorder and Schizophrenia. Front. Psychiatry.

[209] Norwitz N.G., Sethi S., Palmer C.M. (2020). Ketogenic Diet as a Metabolic Treatment for Mental Illness. Curr. Opin. Endocrinol. Diabetes Obes..

[210] Cabeza R., Nyberg L. (2000). Imaging Cognition II: An Empirical Review of 275 PET and fMRI Studies. J. Cogn. Neurosci..

[211] Willette A.A., Bendlin B.B., Starks E.J., Birdsill A.C., Johnson S.C., Christian B.T., Okonkwo O.C., La Rue A., Hermann B.P., Koscik R.L. (2015). Association of Insulin Resistance With Cerebral Glucose Uptake in Late Middle–Aged Adults at Risk for Alzheimer Disease. JAMA Neurol..

[212] Cunnane S., Nugent S., Roy M., Courchesne-Loyer A., Croteau E., Tremblay S., Castellano A., Pifferi F., Bocti C., Paquet N. (2011). Brain Fuel Metabolism, Aging, and Alzheimer’s Disease. Nutrition.

[213] Mujica-Parodi L.R., Amgalan A., Sultan S.F., Antal B., Sun X., Skiena S., Lithen A., Adra N., Ratai E.-M., Weistuch C. (2020). Diet Modulates Brain Network Stability, a Biomarker for Brain Aging, in Young Adults. Proc. Natl. Acad. Sci. USA.

[214] Roy M., Nugent S., Tremblay-Mercier J., Tremblay S., Courchesne-Loyer A., Beaudoin J.-F., Tremblay L., Descoteaux M., Lecomte R., Cunnane S.C. (2012). The Ketogenic Diet Increases Brain Glucose and Ketone Uptake in Aged Rats: A Dual Tracer PET and Volumetric MRI Study. Brain Res..

[215] Xu K., Sun X., Eroku B.O., Tsipis C.P., Puchowicz M.A., LaManna J.C., Takahashi E., Bruley D.F. (2010). Diet-Induced Ketosis Improves Cognitive Performance in Aged Rats. Oxygen Transport to Tissue XXXI.

[216] Jucker M. (2010). The Benefits and Limitations of Animal Models for Translational Research in Neurodegenerative Diseases. Nat. Med..

[217] Alzheimer’s Association (2019). 2019 Alzheimer’s Disease Facts and Figures. Alzheimer’s Dement..

[218] Hoyer S., Oesterreich K., Wagner O. (1988). Glucose Metabolism as the Site of the Primary Abnormality in Early-Onset Dementia of Alzheimer Type?. J. Neurol..

[219] Veech R.L., Chance B., Kashiwaya Y., Lardy H.A., Cahill G.F. (2001). Ketone Bodies, Potential Therapeutic Uses. IUBMB Life.

[220] Cunnane S.C., Courchesne-Loyer A., Vandenberghe C., St-Pierre V., Fortier M., Hennebelle M., Croteau E., Bocti C., Fulop T., Castellano C.-A. (2016). Can Ketones Help Rescue Brain Fuel Supply in Later Life? Implications for Cognitive Health during Aging and the Treatment of Alzheimer’s Disease. Front. Mol. Neurosci..

[221] Castellano C.-A., Nugent S., Paquet N., Tremblay S., Bocti C., Lacombe G., Imbeault H., Turcotte É., Fulop T., Cunnane S.C. (2014). Lower Brain 18F-Fluorodeoxyglucose Uptake but Normal 11C-Acetoacetate Metabolism in Mild Alzheimer’s Disease Dementia. J. Alzheimers Dis..

[222] Croteau E., Castellano C.A., Fortier M., Bocti C., Fulop T., Paquet N., Cunnane S.C. (2018). A Cross-Sectional Comparison of Brain Glucose and Ketone Metabolism in Cognitively Healthy Older Adults, Mild Cognitive Impairment and Early Alzheimer’s Disease. Exp. Gerontol..

[223] Mosconi L., Tsui W.-H., De Santi S., Li J., Rusinek H., Convit A., Li Y., Boppana M., De Leon M.J. (2005). Reduced Hippocampal Metabolism in MCI and AD: Automated FDG-PET Image Analysis. Neurology.

[224] Pearl J. (2022). Causality: Models, Reasoning, and Inference.

[225] Cunnane S.C., Courchesne-Loyer A., St-Pierre V., Vandenberghe C., Pierotti T., Fortier M., Croteau E., Castellano C. (2016). Can Ketones Compensate for Deteriorating Brain Glucose Uptake during Aging? Implications for the Risk and Treatment of Alzheimer’s Disease. Ann. N. Y. Acad. Sci..

[226] Moreno C.L., Mobbs C.V. (2017). Epigenetic Mechanisms Underlying Lifespan and Age-Related Effects of Dietary Restriction and the Ketogenic Diet. Mol. Cell Endocrinol..

[227] Hwang J.-Y., Aromolaran K.A., Zukin R.S. (2017). The Emerging Field of Epigenetics in Neurodegeneration and Neuroprotection. Nat. Rev. Neurosci..

[228] Xu Y., Zheng F., Zhong Q., Zhu Y. (2023). Ketogenic Diet as a Promising Non-Drug Intervention for Alzheimer’s Disease: Mechanisms and Clinical Implications. J. Alzheimers Dis..

[229] Rong L., Peng Y., Shen Q., Chen K., Fang B., Li W. (2024). Effects of Ketogenic Diet on Cognitive Function of Patients with Alzheimer’s Disease: A Systematic Review and Meta-Analysis. J. Nutr. Health Aging.

[230] Lilamand M., Mouton-Liger F., Di Valentin E., Sànchez Ortiz M., Paquet C. (2022). Efficacy and Safety of Ketone Supplementation or Ketogenic Diets for Alzheimer’s Disease: A Mini Review. Front. Nutr..

[231] Gabuzyan R., Lee C., Nygaard H.B. (2024). Ketogenic Approaches for the Treatment of Alzheimer’s Disease. J. Alzheimers Dis..

[232] McKay N.S., Gordon B.A., Hornbeck R.C., Dincer A., Flores S., Keefe S.J., Joseph-Mathurin N., Jack C.R., Koeppe R., Millar P.R. (2023). Positron Emission Tomography and Magnetic Resonance Imaging Methods and Datasets within the Dominantly Inherited Alzheimer Network (DIAN). Nat. Neurosci..

[233] Yassine H.N., Self W., Kerman B.E., Santoni G., Navalpur Shanmugam N., Abdullah L., Golden L.R., Fonteh A.N., Harrington M.G., Gräff J. (2023). Nutritional Metabolism and Cerebral Bioenergetics in Alzheimer’s Disease and Related Dementias. Alzheimers Dement..

[234] Fante C., Spritzler F., Calabrese L., Laurent N., Roberts C., Deloudi S. (2025). The Role of β-Hydroxybutyrate Testing in Ketogenic Metabolic Therapies. Front. Nutr..

[235] O’Neill B., Raggi P. (2020). The Ketogenic Diet: Pros and Cons. Atherosclerosis.

[236] Bougnères P.F., Saudubray J.M., Marsac C., Bernard O., Odievre M., Girard J.R. (1980). Decreased Ketogenesis Due to Deficiency of Hepatic Carnitine Acyl Transferase. N. Engl. J. Med..

